# The signatures and crosstalk of gut microbiome, mycobiome, and metabolites in decompensated cirrhotic patients

**DOI:** 10.3389/fmicb.2024.1443182

**Published:** 2024-08-21

**Authors:** Yangjie Li, Danping Liu, Yanglan He, Zeming Zhang, Ajuan Zeng, Chunlei Fan, Lingna Lyu, Zilong He, Huiguo Ding

**Affiliations:** ^1^Department of Gastroenterology and Hepatology, Laboratory for Clinical Medicine, Beijing You'an Hospital Affiliated to Capital Medical University, Beijing, China; ^2^School of Engineering Medicine, Beihang University, Beijing, China; ^3^Key Laboratory of Big Data-Based Precision Medicine, Beihang University, Ministry of Industry and Information Technology of the People's Republic of China, Beijing, China; ^4^Key Laboratory of Biomechanics and Mechanobiology, Beihang University, Ministry of Education, Beijing, China

**Keywords:** cirrhosis, gut dysbiosis, bacteria, fungi, metabolome

## Abstract

**Background:**

Numerous studies have confirmed that gut microbiota plays a crucial role in the progression of cirrhosis. However, the contribution of gut fungi in cirrhosis is often overlooked due to the relatively low abundance.

**Methods:**

We employed 16S ribosomal RNA sequencing, internal transcribed spacer sequencing, and untargeted metabolomics techniques to investigate the composition and interaction of gut bacteria, fungi, and metabolites in cirrhotic patients.

**Results:**

Cirrhotic patients exhibited significant differences in the diversity and composition of gut microbiota and their metabolites in cirrhotic patients compared to healthy individuals. Increase in pathogenic microbial genera and a decrease in beneficial microbial genera including bacteria and fungi were observed. Various clinical indexes were closely connected with these increased metabolites, bacteria, fungi. Additionally, endoscopic treatment was found to impact the gut microbiota and metabolites in cirrhotic patients, although it did not significantly alter the gut ecology. Finally, we constructed a cirrhosis diagnostic model based on different features (bacteria, fungi, metabolites, clinical indexes) with an AUC of 0.938.

**Conclusion:**

Our findings revealed the characteristics of gut microbial composition and their intricate internal crosstalk in cirrhotic patients, providing cutting-edge explorations of potential roles of gut microbes in cirrhosis.

## Introduction

Human gastrointestinal tracts harbor trillions of microbes (about 10^12^-10^14^), including bacteria, fungi, archaea, and viruses, which are involved in many physiological functions and disease pathogenesis (Gomaa, 2020). In recent years, the importance of intestines to human health and dysregulation of the gut microbes in different diseases has been implicated based on high-throughput sequencing technology (Lynch et al., 2016). Due to its unique anatomical location, the liver is regarded as the first organ to encounter gut microbes and their products through the gut epithelial barrier. The blood supply to the liver comes from the hepatic artery and portal vein. Portal blood contributes to 75% of the total blood flow to the liver and transfers nutrients, enteric-borne microbes, and pathogen-associated molecular patterns to liver originating from the intestine (Llorente and Schnabl, 2015; Hsu and Schnabl, 2023). Thus, the liver and gastrointestinal tract are interconnected, known as the gut-liver axis (Tripathi et al., 2018). Appreciable evidence suggests that gut microbes play a central role in the gut-liver axis and are closely related to liver diseases (Schnabl and Brenner, 2014; Pabst et al., 2023). However, the contributions of gut microbes to the occurrence and progression of liver diseases and the feasibility of clinical applications targeting gut microbes have yet to be fully elucidated.

Liver cirrhosis (LC) is a common outcome of chronic liver diseases, characterized by tissue fibrosis and the transformation of normal liver architecture into abnormal nodules (Kisseleva and Brenner, 2020). LC progression eventually leads to the development of portal hypertension and its associated complications with high mortality, accounting for 1.2 million deaths per year and nearly 3.5% of global mortality (Moon et al., 2020). The gut microbes have been proven to be related to the induction and progression of liver injury (Yan et al., 2011). Gut bacterial dysbiosis may adversely disorder liver homeostasis, leading to the progression and complications of LC (Qin et al., 2014; Lachar and Bajaj, 2016). Accumulating studies have revealed significant microbial dysbiosis with substantially decreased richness, diversity, and altered composition of the gut bacteria translocation in LC patients compared with healthy individuals (Bajaj et al., 2014; Shao et al., 2018).

In addition, bacterial and fungal communities do have significant impacts on the homeostasis of the intestinal microecosystem, although colonizing fungi account for a minor component of the gut microbiota (Dworecka-Kaszak et al., 2016). Traditional culture-dependent methods can only detect small proportions of fungi. With the development of high-throughput sequencing technology, fungal sequencing methods, including 18S ribosomal RNA sequencing and fungal-specific internal transcribed spacer DNA sequencing, have gradually replaced traditional methods for studying fungi. With the growing knowledge of the characteristics and species of fungi, complex relationships between gut fungi and human health were discovered, and the alterations of fungal communities may contribute to the occurrence of liver diseases (Zeng and Schnabl, 2022). Clinical studies suggested that fungal colonization and translocation could increase the mortality rate of LC patients in intensive care unit (ICU; Theocharidou et al., 2016; Verma et al., 2022). Consistently, a cross-sectional study has revealed the presence of dysregulated intestinal fungi in cases of cirrhosis and hepatocellular carcinoma by ITS (internal transcribed spacer) DNA sequencing (Bajaj et al., 2018; Zhang et al., 2023). Although bacteria and fungi are dysregulated in cirrhosis, previous efforts mainly focused on bacteria, the impact of the differences in fungi and the crosstalk between fungi and bacteria in patients with cirrhosis is barely known, especially in those with endoscopic treatment.

In the present study, we performed 16S ribosomal RNA and ITS sequencing, liquid chromatography coupled to tandem mass spectrometry (LC-MS/MS) technology to reveal gut microbial characteristics and explore the connections among intestinal bacteria, fungi, and metabolites in patients with liver cirrhosis. Our study reveals the complex interactions within the intestinal microbiota of cirrhotic patients, providing new insights for the diagnosis and treatment of liver cirrhosis.

## Materials and methods

### Patients and sample collection

A total of 45 patients with decompensated liver cirrhosis who were admitted to the Department of Gastroenterology and Hepatology of Beijing You'an Hospital affiliated with Capital Medical University from January to May 2023 were enrolled, while another 30 healthy people without any chronic diseases were selected as control group. Patients were diagnosed with liver cirrhosis in terms of clinical symptoms, medical history, laboratory tests, imaging tests, or histological examinations, and complications included esophagogastric varices (EGV), ascites, hepatic encephalopathy (HE), portal vein thrombosis (PVT). The demographic and clinical data of the subjects involved were collected through the electronic medical record system. Exclusion criteria included the following conditions: aged < 18 or >75; ursodeoxycholic acid, probiotics, or antibiotics treatment before sample collection; hepatic encephalopathy > grade 2 and/or other cognitive disorders not allowing for informed consent; suffered from malignant cancers including hepatocellular carcinoma, severe cardiopulmonary failure, and other diseases (gastrointestinal intestinal polyps, inflammatory bowel disease, necrotizing enteritis, diabetes, etc.). Feces samples were collected in the morning after an overnight fast, delivered to the laboratory within 2 h on dry ice, and stored at −80°C until further analyses. In addition, to further explore the short-term effects of endoscopic treatment of esophagogastric variceal on the gastrointestinal flora, we also collected the feces of patients who underwent endoscopic treatment 10–14 days. The Ethics Committee of Beijing You'an Hospital authorized the project (LL-2022-065-K), and Informed consent was obtained from all individual participants included in the study.

### Clinical data analysis

Descriptive and comparative statistics were used to assess and compare clinical data between two groups. Quantitative data were represented as Mean ± SE (standard error) and categorical variables were represented by the number of cases and percentage. Continuous data was performed by using *t***-**test, the chi-square test and the Fisher's precision probability test for categorical variables. *P***-**value < 0.05 was considered statistically significant.

### DNA extraction, PCR amplification, and sequencing

Genomic DNA was extracted using the DNeasy Power Water DNA Isolation Kit (Qiagen, Germany, Cat: 14900-100-NF) according to the manufacturer's instructions with minor modifications for separate extraction of bacterial and fungal genomic DNA.

Equal concentrations of bacterial and fungal DNA (~10 ng) were used for PCR amplification of the bacterial 16S rRNA V4 hypervariable region and fungal ITS2 region. The V4 primers are forward 5**′** -GTGCCAGCMGCCGCGGTAA-3**′** and reverse 5**′**-GGACTACHVGGGTWTCTAAT-3**′** (Sun et al., 2013). For fungi, the ITS2 intergenic region were amplified using the forward primer (5**′**-GCATCGATGAAGAACGCAGC-3**′**) and reverse (5**′**-TCCTCCGCTTATTGATATGC-3**′**; Zhang et al., 2023). 16S rRNA V4 hypervariable region and ITS2 region were amplified used the specific primer with the barcode. Sequencing libraries were generated using Illumina TruSeq DNA PCR-Free Library Preparation Kit (Illumina, USA) following manufacturer's recommendations and index codes were added. The library quality was assessed on the Qubit^@^ 2.0 Fluorometer (Thermo Scientific) and Agilent Bioanalyzer 2100 system. At last, the library was sequenced on an Illumina NovaSeq platform and 250 bp paired-end reads were generated.

### Bioinformatics and statistical analysis

Raw bacterial and fungal amplicon sequences were processed and analyzed with the EasyAmplicon (Liu et al., 2023) pipeline (version 1.19, https://github.com/YongxinLiu/EasyAmplicon). First, FastQC (version 0.12.1) was used to control the raw data quality and confirm the absence of primers. Subsequently, the software VSEARCH (Rognes et al., 2016; version 2.22.1) was used to merge pair-end reads (-fastq_mergepairs), filter (-fastq_maxee_rate 0.01), and dereplicate (-derep_fulllength). The obtained high-quality, unique sequences were denoised into amplicon sequence variants (ASVs) with the software USEARCH (Edgar, 2010; version 11.0.667). Chimeras were identified and removed from the bacterial and fungal data using VSEARCH(-uchime_ref) against the SILVA (Quast et al., 2012) v123 database and UNITE (Abarenkov et al., 2023) v9.0 database, respectively. Finally, based on the chimera-free ASVs, we constructed feature tables using VSEARCH (-usearch_global). Species classification of bacterial and fungal ASVs was done based on the RDP (Cole et al., 2014) v18 database and UNITE v9.0 database, with USEARCH (-sintax), respectively. Bacterial and fungal sequences of all samples were rarefied to 62,889 and 5,766 for downstream analysis, respectively.

Alpha diversity of bacterial and fungal communities was calculated using USEARCH (-alpha_div) based on the richness index. The differences between groups were assessed using Tukey's HSD test. Beta diversity was calculated using USEARCH (-cluster_agg, -beta_div) through principal coordinate analysis (PCoA) based on the Bray-Curtis distance. Permutational multivariate analysis of variance (PERMANOVA) with the ADONIS test was used to assess the differences between groups. Linear discriminant analysis (LDA) effect size (LEfSe) was used to determine biomarkers at the genus level between groups (LDA score>3). The functional composition of bacterial and fungal communities was predicted based on the MetaCyc (Caspi et al., 2018) database (https://metacyc.org/) using Phylogenetic Investigation of Communities by Reconstruction of Unobserved States (PICRUSt2; Douglas et al., 2020) software. The *P***-**values of metabolic pathways differing between groups were calculated by Welch's *t*-test and corrected using the Benjamini-Hochberg false discovery rate (FDR). Orthogonal partial least squares discriminant analysis (OPLS-DA) was used to determine the differences in metabolite composition among samples. The screening criteria for differential metabolites were: *P***-**value of Student's *t*-test < 0.05, Variable Importance in the Projection (VIP) values obtained from the OPLS -DA model was >1, and the absolute value of log_2_(fold change) was >0. The VIP values were used to assess the contribution of the different metabolites to the differences between groups. Pathway enrichment analysis of differential metabolites was done based on the Kyoto Encyclopedia of Genes and Genomes (Kanehisa, 2000) pathway database. Metabolic pathways that were significantly enriched (*P***-**value < 0.05) were identified by the hypergeometric test.

Associations among differential bacteria-clinical indexes, differential fungi-clinical indexes, and differential metabolite-clinical indexes, as well as bacteria-fungi-differential metabolites in liver cirrhotic patients, were determined based on the Spearman's correlation analysis (correlation coefficient > 0.4, *P***-**value < 0.05). The random forest model used to distinguish liver cirrhosis patients from healthy individuals was constructed using the R package “randomForest.” The specific construction process of the model is as follows. The first step is to determine the training and test sets. The training set consisted of 34 liver cirrhosis patients and 23 healthy individuals. The test set consisted of 11 liver cirrhosis patients and seven healthy individuals. Random forest models were then constructed using the training set (ntree = 1,000) and the importance of different features (bacteria, fungi, metabolites or clinical indexes) was evaluated (importance = TRUE). Subsequently, the optimal number of features to use was determined based on the 10-fold cross-validation results and models were further optimized to select the best mtry value. Finally, the prediction accuracy was tested in the test set and area under curve (AUC) values were calculated. All above Bioinformatic analysis results were visualized using the Wekemo Bioincloud (Gao et al., 2024; https://www.bioincloud.tech), ImageGP (Chen T. et al., 2022), STAMP (Parks et al., 2014; version 2.1.3), R packages “ggplot2” (https://ggplot2.tidyverse.org), “circlize” (http://cran.r-project.org/web/packages/circlize/), and “pheatmap” (https://cran.r-project.org/web/packages/pheatmap/).

### Untargeted metabolomic data preparation and analysis

100 μL of sample was taken, mixed with 400 μL of extraction solution [MeOH: ACN, 1:1 (v/v)], the extraction solution contains deuterated internal standards, the mixed solution was vortexed for 30 s, sonicated for 10 min in 4°C water bath, and incubated for 1 h at −40°C to precipitate proteins. Then the samples were centrifuged at 12,000 rpm (RCF = 13,800 (× g), *R*= 8.6 cm) for 15 min at 4°C. The supernatant was transferred to a fresh glass vial for analysis. The quality control (QC) sample was prepared by mixing an equal aliquot of the supernatant of samples.

LC-MS/MS analyses were performed using an UHPLC system (Vanquish, Thermo Fisher Scientific) with a Waters ACQUITY UPLC BEH Amide (2.1 × 50 mm, 1.7 μm) coupled to Orbitrap Exploris 120 mass spectrometer (Orbitrap MS, Thermo). The mobile phase consisted of 25 mmol/L ammonium acetate and 25 mmol/L ammonia hydroxide in water (pH = 9.75) (A) and acetonitrile (B). The auto-sampler temperature was 4°C, and the injection volume was 2 μL. The Orbitrap Exploris 120 mass spectrometer was used for its ability to acquire MS/MS spectra on information-dependent acquisition (IDA) mode in the control of the acquisition software (Xcalibur, Thermo). In this mode, the acquisition software continuously evaluates the full scan MS spectrum. The ESI source conditions were set as following: sheath gas flow rate as 50 Arb, Aux gas flow rate as 15 Arb, capillary temperature 320°C, full MS resolution as 60,000, MS/MS resolution as 15,000, collision energy: SNCE 20/30/40, spray voltage as 3.8 kV (positive) or −3.4 kV (negative), respectively.

The raw data were converted to the mzXML format using ProteoWizard and processed with an in-house program, which was developed using R and based on XCMS, for peak detection, extraction, alignment, and integration. Then a MS2 database was applied in metabolite annotation. The cutoff for annotation was set at 0.3.

## Results

### Clinical characteristics of participants

A total of 75 participants were included in this study: 45 liver cirrhotic patients and 30 healthy controls. The baseline clinical and demographic data for all groups were shown in Table 1. Age (*P* = 0.067) and gender (*P* = 0.745) were matched without significant differences between LC and C groups. Serum levels of ALT, TBIL, PT, and INR were elevated, whereas WBC, HB, PLT, ALB, and PTA were decreased in the LC group compared with healthy controls (*P* < 0.05). In cirrhotic patients, the median CTP scores and MELD scores were five and nine, respectively. The distribution of etiologies of LC was as follows: HBV/HCV, 25 patients; ALD, ten patients; NAFLD, five patients; AIH, five patients. Additionally, 15 patients were accompanied by EGV complications and underwent endoscopic treatment.

**Table 1:** Demographics and clinical characteristics of subjects.

**Clinical variables**	**LC (*n* = 45)**	**C (*n* = 30)**	***t*/χ^2^-value**	***P*-value**
Age	56.2 ± 1.6	51.6 ± 2.0	−1.86	0.067
Gender, male, *n* (%)	33 (73.3)	23 (76.7)	0.11	0.745
WBC (10^9^/L)	3.3 ± 0.3	6 ± 0.3	6.53	< 0.001
Hb (g/L)	110 ± 4.2	153 ± 2.6	8.61	< 0.001
PLT (10^9^/L)	95.9 ± 13.3	240 ± 11.3	7.67	< 0.001
ALT (U/L)	22.5 ± 1.7	27.6 ± 2.7	1.68	0.097
AST (U/L)	31.8 ± 2	23.9 ± 1.3	−3.31	0.001
TBIL (μmol/L)	22 ± 1.9	15.2 ± 0.8	−3.21	0.002
ALB (g/L)	36.8 ± 0.7	43.4 ± 1.5	4.54	< 0.001
PT (s)	11.3 ± 0.3	10.2 ± 0.1	3.40	0.001
PTA (%)	66.27 ± 1.8	103.3 ± 1.4	14.96	< 0.001
INR	1.2 ± 0.2	1 ± 0.2	−5.07	< 0.001
CTP scores	5 (5, 7)	-	-	-
MELD scores	9 (8,11)	-	-	-
Etiology		-	-	-
HBV/HCV	25 (55.6)	-	-	-
ALD	10 (22.2)	-	-	-
NAFLD	5 (11.1)	-	-	-
AIH	5 (11.1)	-	-	-
Complications				
EGV, *n* (%)	15 (33.3)	-	-	-
PVT, *n* (%)	11 (24.4)	-	-	-
HE, *n* (%)	2(4.4%)	-	-	-
Ascites, *n* (%)	9 (20)	-	-	-

LC, liver cirrhosis; C, healthy controls; WBC, white blood cell; Hb, hemoglobin; PLT, blood platelet; ALT, alanine transaminase; AST, aspartate aminotransferase; TBIL, total bilirubin; PT, prothrombin time; PTA, prothrombin activity; INR, international normalized ratio; CTP scores, Child-Turcotte-Pugh scores; MELD scores, Model for End-Stage Liver Disease scores; HBV/HCV, Hepatitis B/C Virus; ALD, alcoholic liver disease; NAFLD, non-alcoholic fatty liver disease; AIH, autoimmune liver disease; EGV, esophageal and gastric variceal; PVT, portal vein emboli; HE, hepatic encephalopathy.

### Characterization of the intestinal microbiome and mycobiome in decompensated liver cirrhotic patients

**Figure 1:**
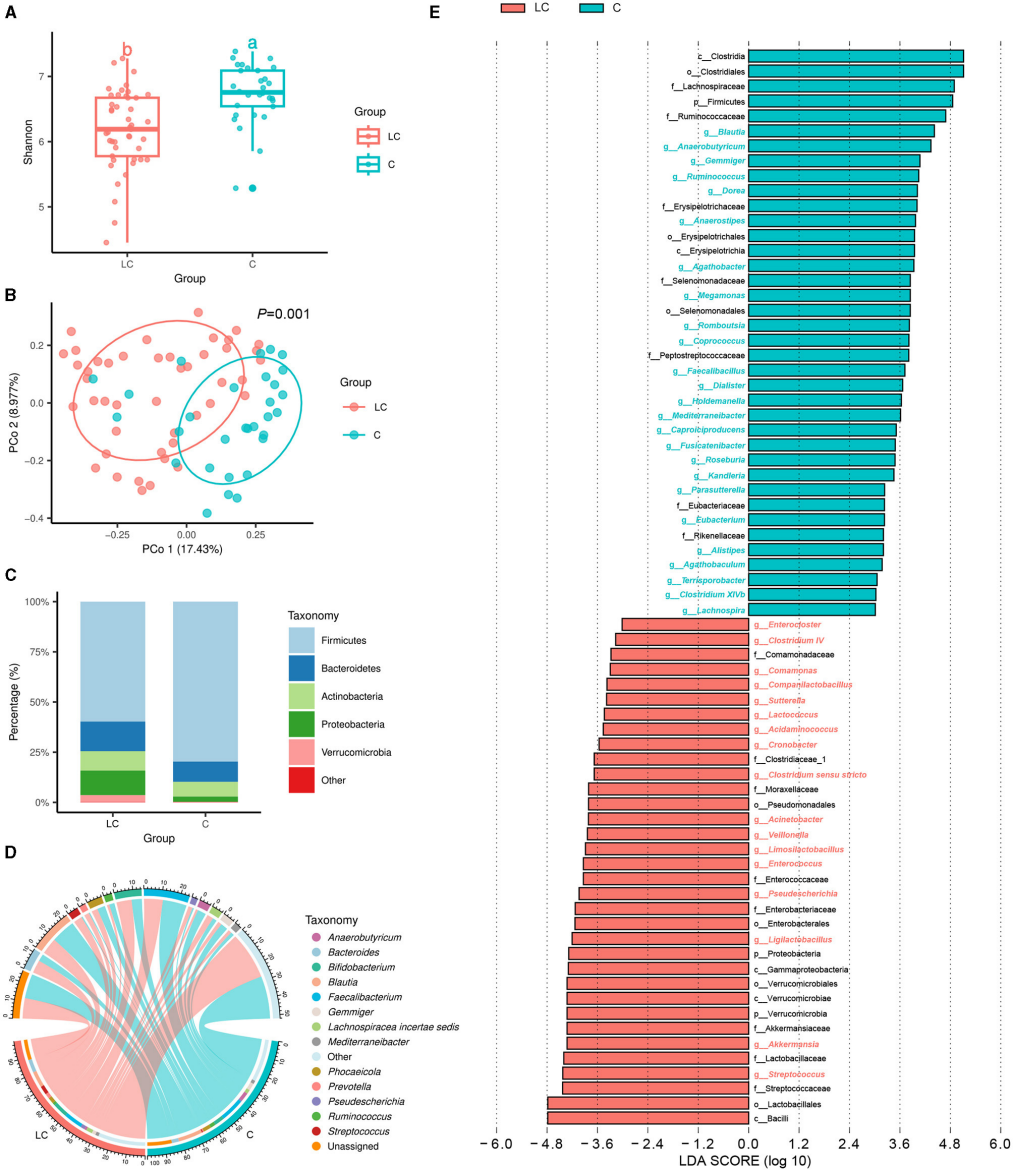
Characterization of intestinal bacterial communities in liver cirrhosis patients. **(A)** Alpha diversity of bacterial communities between groups based on Shannon index. **(B)** Principal coordinate analysis (PCoA) based on Bray-Curtis distance. **(C)** Bacterial community composition (phylum level). **(D)** Bacterial community composition (genus level). **(E)** LEfSe analysis reveals differential bacteria between groups (LDA score > 3).

A total of 10,274,244 high-quality 16S rRNA reads were obtained from fecal samples of 45 patients with liver cirrhosis (LC group) and 30 healthy individuals (C group). After sequence processing, the final number of 6,861 ASVs was obtained. The rarefaction curve of richness indicates that the current sample was sequenced at a reasonable depth (Supplementary Figure 1A). One hundred and twenty-five and 102 ASVs were specific to cirrhotic patients and healthy individuals, respectively (Supplementary Figure 1B). In terms of alpha diversity, the Shannon index was significantly lower in cirrhotic patients than in healthy individuals (*P* < 0.01, Tukey's HSD test; Figure 1A). In terms of beta diversity, the results of principal coordinate analysis (PCoA) based on Bray-Curtis distance showed that the gut bacterial community structure of cirrhotic patients was significantly different from that of healthy individuals (*P* = 0.001, PERMANOVA with ADONIS test; Figure 1B). After alignment with the RDP database, 6,861 ASVs were annotated to a total of 6 phyla, 12 classes, 19 orders, 33 families, and 83 genera. On average, more than half of the bacteria in different subgroups were from Firmicutes (Figure 1C). *Faecalibacterium, Blautia*, and *Bifidobacterium* were the three genera with the highest average relative abundance (Figure 1D). Subsequently, LEfSe was used to further identify bacterial genera with significant differences in abundance across subgroups (Figure 1E). There were 17 and 25 bacterial genera enriched (LDA score > 3) in cirrhotic patients and healthy individuals, respectively. Among them, *Streptococcus, Akkermansia, Ligilactobacillus, Pseudescherichia* were signifantly enriched in liver cirrhotic patients, while *Blautia, Anaerobutyricum, Gemmiger, Ruminococcus*, and *Dorea* were markedly decresed (LDA score >4). In addition, the functional composition of the bacterial communities was enriched in bacterial-associated metabolic pathways (Supplementary Figure 1C). Several metabolic pathways differed significantly from LC and healthy individuals (Supplementary Figure 1D).

**Figure 2:**
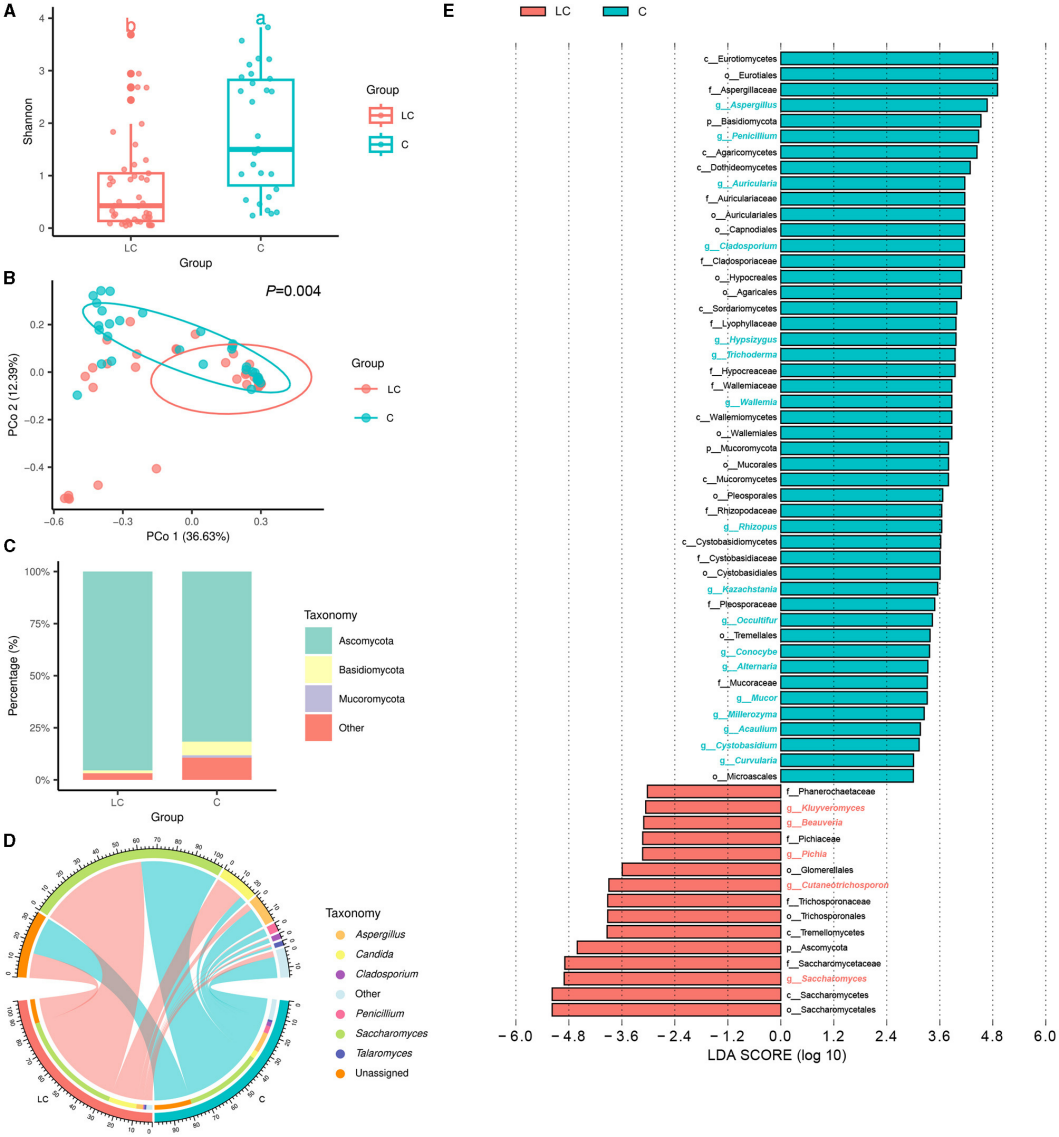
Characterization of intestinal fungal communities in liver cirrhosis patients. **(A)** Alpha diversity of fungal communities based on Shannon index. **(B)** Principal coordinate analysis (PCoA) based on Bray-Curtis distance. **(C)** Fungal community composition (phylum level). **(D)** Fungal community composition (genus level). **(E)** LEfSe analysis reveals differential fungi between groups (LDA score > 3).

In terms of gut mycobiome, a total of 9,594,331 high-quality ITS rRNA reads were obtained from fecal samples of 45 cirrhotic patients and 30 healthy individuals. After sequence processing, the final number of ASVs was 901. The rarefaction curve of richness indicates that the current sample was sequenced at a reasonable depth (Supplementary Figure 2A). There were 18 and 60 ASVs specific to cirrhotic patients and healthy individuals, respectively (Supplementary Figure 2B). The Shannon index was significantly lower in cirrhotic patients than in healthy individuals (*P* < 0.01, Tukey's HSD test; Figure 2A). The results of PCoA showed that the gut fungal community structure of cirrhotic patients was significantly different from that of healthy individuals (*P* = 0.004, PERMANOVA with ADONIS test; Figure 2B). After alignment with the UNITE database, 901 ASVs were annotated to a total of 8 phyla, 20 classes, 51 orders, 101 families, 116 genera, and 125 species. On average, more than 80% of the fungi in the different subgroups were from Ascomycota (Figure 2C). *Saccharomyces, Candida*, and *Aspergillus* were the three genera with the highest average relative abundance (Figure 2D). Subsequently, LEfSe analysis indicated that the *Saccharomyces* was significantly increased and *Aspergillus, Penicillium, Auricularia*, as well as *Cladosporium* were reduced in cirrhotic patients (LDA score >4). In addition, we analyzed the functional composition of the fungal communities (Supplementary Figure 2C) and predicted 69 fungal-related metabolic pathways. There were 15 metabolic pathways with mean relative abundance differences >0.3% between groups, of which six were enriched in liver cirrhotic patients, including aerobic respiration II (cytochrome c; yeast), aerobic respiration I (cytochrome c), pentose phosphate pathway, glycolysis III (from glucose), CDP-diacylglycerol biosynthesis I, and adenosine ribonucleotides *de novo* biosynthesis (Supplementary Figure 2D).

### Characterization of the intestinal metabolome in liver cirrhotic patients

**Figure 3:**
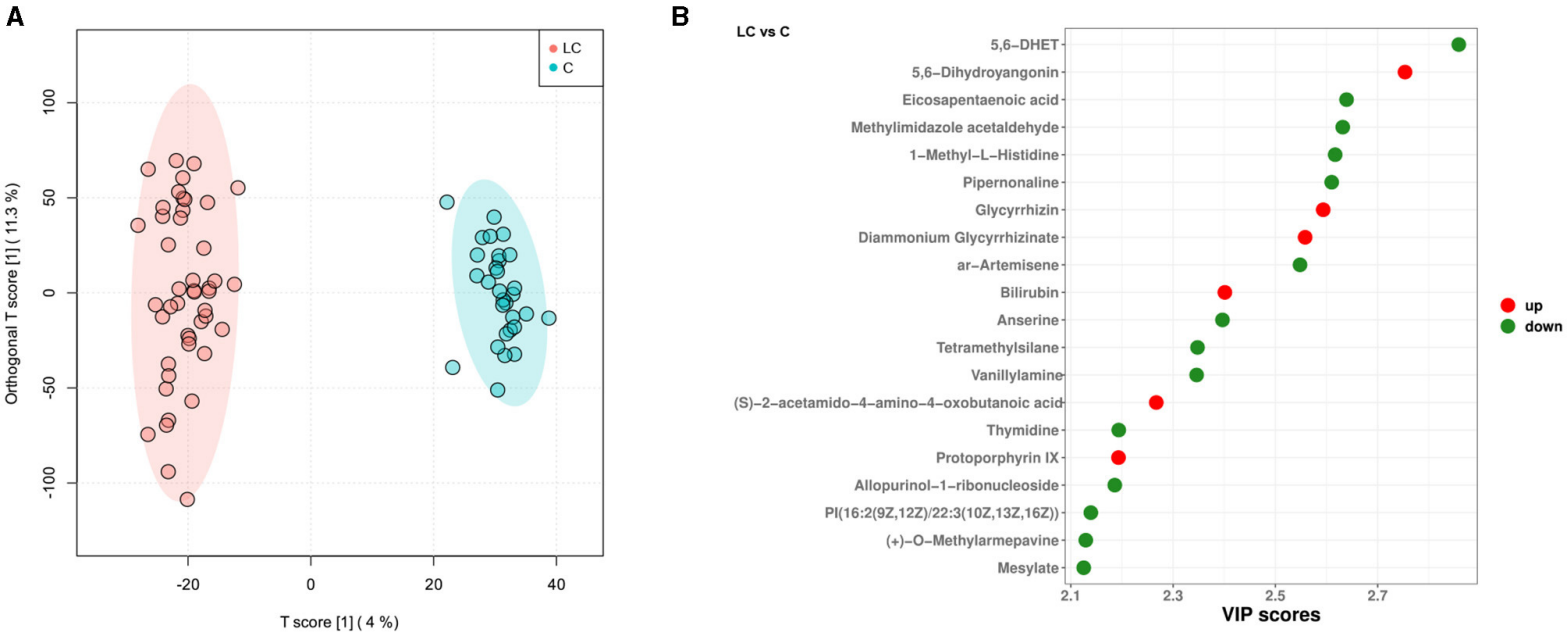
Characterization of intestinal metabolites in liver cirrhosis patients. **(A)** OPLS-DA analysis reveals differences in metabolite composition of samples between groups. Red color represents 45 liver cirrhosis patients (LC group) and blue color represents 30 healthy individuals (C group). **(B)** Top20 intergroup differential metabolites (sorted by VIP score).

Based on the untargeted metabolomic technology, we detected 11,382 variables in fecal samples from 45 cirrhotic patients and 30 healthy individuals. Of these, 727 variables were enriched in cirrhotic patients [VIP score>1, *P* < 0.05, |log_2_(fold change) |>0], and 814 variables were enriched in healthy individuals (Supplementary Figure 3A). OPLS-DA analysis similarly demonstrated significant differences in the metabolite composition of the samples from different subgroups (Figure 3A). After metabolite annotation, we finally identified 130 differential metabolites. Sixty-eight of these metabolites were enriched in cirrhotic patients, and another 62 were enriched in healthy individuals (Supplementary Figure 3B). Ranking all differential metabolites by VIP score, among the top 20 differential metabolites: 5,6-dihydroyangonin, glycyrrhizin, diammonium glycyrrhizinate, bilirubin, (S)-2-acetamido-4-amino-4-oxobutanoic acid, and protoporphyrin IX were enriched in cirrhotic patients (Figure 3B). To further explore the biological processes in which the differential metabolites might be involved, we performed metabolic pathway annotation and enrichment analysis based on the KEGG PATHWAY Database. The results revealed that 49 differential metabolites were annotated to 75 metabolic pathways. Among them, the “porphyrin metabolism” pathway showed the most significant enrichment effect (*P* = 0.053, hypergeometric test; Supplementary Figure 3C). Bilirubin, biliverdin, protoporphyrin IX, and coproporphyrin I were jointly involved in this metabolic pathway.

### The effects of endoscopic treatment on gut bacteria, fungi, and metabolites

**Figure 4:**
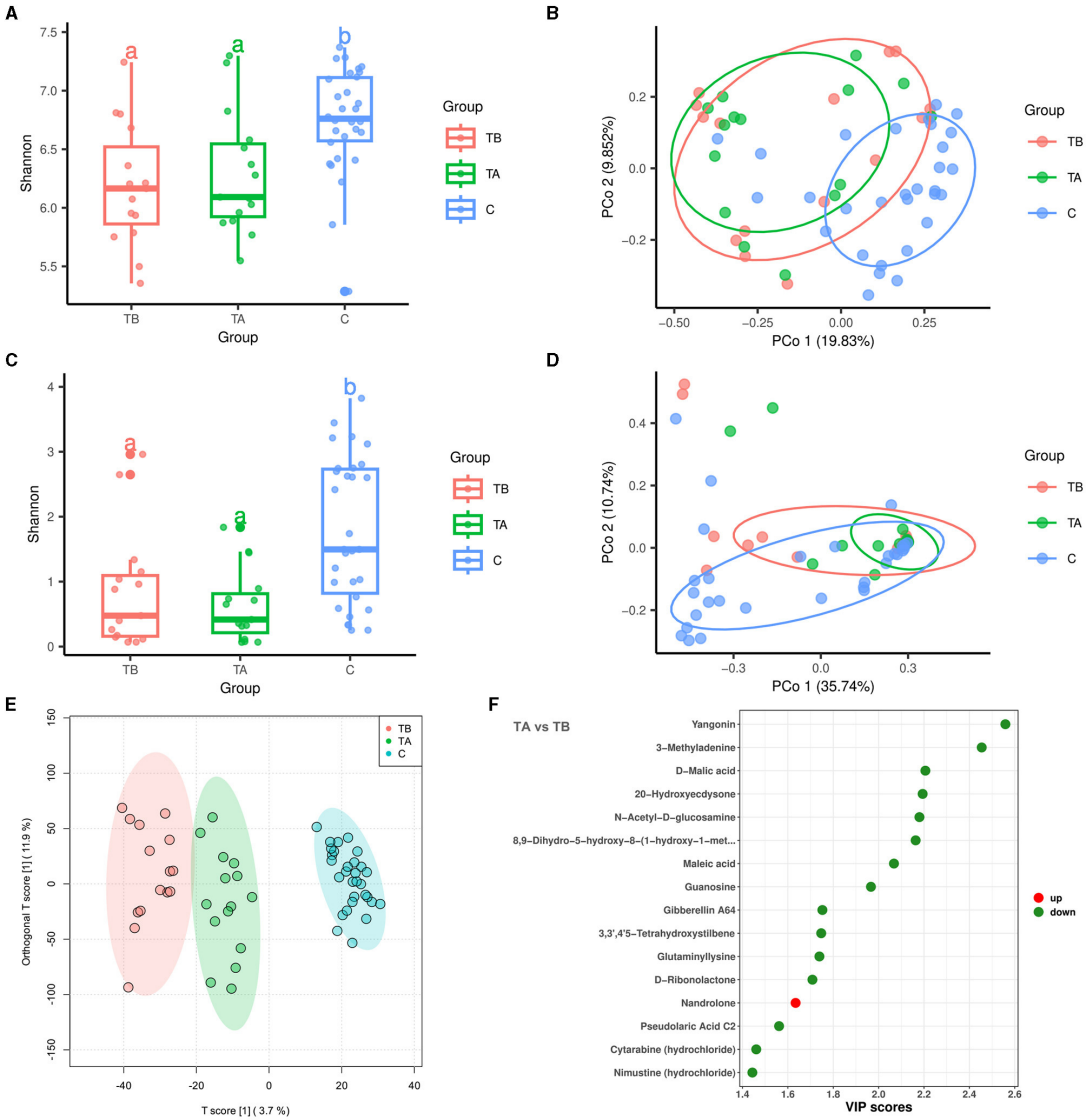
Effect of endoscopic treatment on intestinal bacteria, fungi, and metabolites in liver cirrhosis patients. Red, green, and blue colors represent 15 treatment-before cirrhotic patients (TB group), 15 treatment-after cirrhotic patients (TA group), and 30 healthy individuals (C group), respectively. **(A)** Alpha diversity of bacterial communities based on Shannon index. **(B)** Principal coordinate analysis (PCoA; bacteria) based on Bray-Curtis distance (*P* = 0.001, PERMANOVA with ADONIS test). **(C)** Alpha diversity of fungal communities based on Shannon index. **(D)** Principal coordinate analysis (PCoA; fungi) based on Bray-Curtis distance. **(E)** OPLS-DA analysis reveals differences in metabolite composition of samples between groups. **(F)** Differential metabolites in patients with cirrhosis before and after treatment (sorted by VIP score).

In this study, 15 cirrhotic patients had fecal samples collected before and after undergoing the endoscopic treatment. Based on this, we further explored the effects of endoscopic treatment on the intestinal bacteria, fungi, and metabolome of cirrhotic patients. A total of 8,506,082 high-quality 16S rRNA reads were obtained from fecal samples of 15 treatment-before cirrhotic patients (TB group), 15 treatment-after cirrhotic patients (TA group), and 30 healthy individuals (C group). A total of 8,009,700 high-quality ITS rRNA reads were obtained. The average number of reads per sample was 133,495 (5,771–280,279). After sequence processing, 7,069 ASVs (bacteria) and 712 ASVs (fungi) were obtained. The rarefication curve of richness indicated that the current sample was sequenced at a reasonable depth (Supplementary Figures 4A, E). Compared with the TB group, the alpha diversity and beta diversity of the TA group did not change significantly for either bacterial or fungal communities (Figures 4A–D). Species classification results of bacteria and fungi showed that at the phylum and genus levels, the TA group had a similar species composition as the TB group (Supplementary Figures 4B, C, F, G). LEfSe results showed that the relative abundance of several bacterial and fungal genera changed significantly after endoscopic treatment (Supplementary Figures 4D, H). Among them, the bacterial genus *Acinetobacter* and the fungal genus *Cutaneotrichosporon* (LDA score >4) were enriched in the TB group, while the bacterial genus *Megamonas* (LDA score > 4) and the fungal genera *Coniochaeta* and *Ascotricha* (LDA score > 3) were decreased. In contrast, no differential metabolic pathways related to bacteria and fungi were found in predicting community functional composition based on the MetaCyc database using PICRUSt2 software.

A total of 11,156 variables were detected in the metabolomic analysis of 15 before- and 15 after-treatment cirrhotic patients. Of these, 43 variables were enriched in the TA group [VIP score > 1, *P* < 0.05, |log_2_(fold change) |>0], and 192 variables were enriched in the TB group (Supplementary Figure 4I). The OPLS-DA analysis demonstrated that before and after endoscopic treatment, samples showed significant differences in metabolite compositions (Figure 4E). After metabolite annotation, we finally identified 16 differential metabolites. Only nandrolone was enriched in the TA group, and the other 15 metabolites were enriched in the TB group (Supplementary Figure 4J, Figure 4F). Metabolic pathway annotation and enrichment analysis based on the KEGG PATHWAY Database showed that four differential metabolites were annotated to seven metabolic pathways. Among them, the “butanoate metabolism” pathway showed the most significant enrichment effect (*P* = 0.0058, hypergeometric test; Supplementary Figure 4K). Maleic acid and D-malic acid (enriched in the TB group) were involved in this metabolic pathway.

### Multi-omics integration analysis

**Figure 5:**
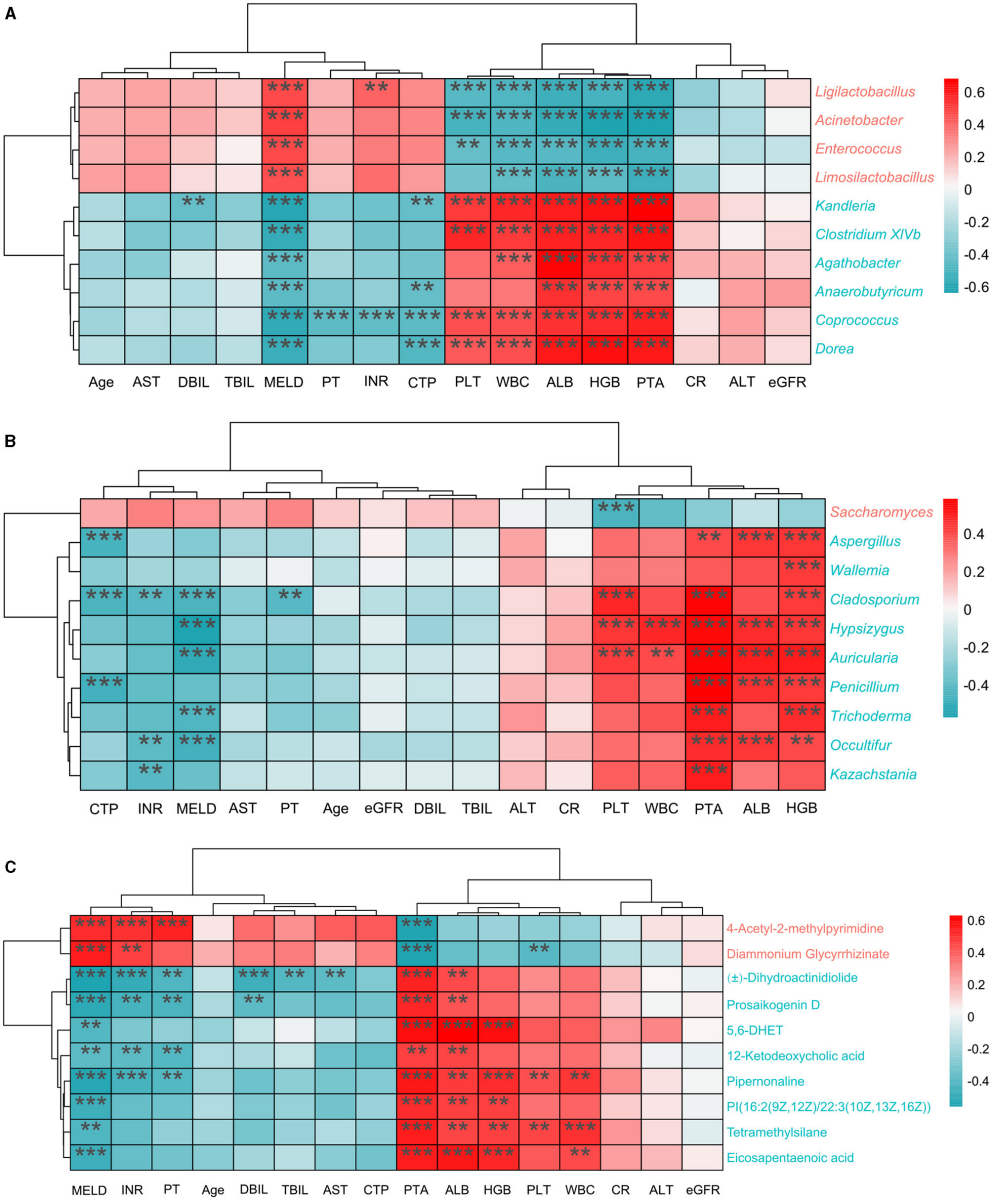
Correlation analysis of clinical indexes with different features. **(A)** Top10 differential bacterial genera most correlated with clinical indexes. **(B)** Top10 differential fungal genera most correlated with clinical indexes. **(C)** Top10 differential metabolites most correlated with clinical indexes. The red color in the heatmap represents positive correlation and the blue color represents negative correlation. *P* < 0.05; **P < 0.01; ***P < 0.001 (FDR corrected).

To explore the association between clinical indexes and differential bacteria, fungi, and metabolites, we performed the Spearman's correlation analysis based on 45 cirrhotic patients and 30 healthy individuals. Depending on the number of significant factors and the size of the *p*-values, we finally demonstrated the ten most highly correlated features (differential bacteria, fungi, and metabolites) in heatmaps (Figure 5). Overall, there were significant associations between the clinical indexes ALB, HGB, MELD, PLT, PTA, and WBC with various features. Among bacterial genera, *Acinetobacter* (enriched in cirrhotic patients) significantly negatively correlated with HGB and PTA indexes (correlation coefficient < -0.6, *P* < 0.001). *Dorea* (enriched in healthy individuals) significantly positively correlated with ALB, HGB, and PTA indexes (correlation coefficient>0.6, *P* < 0.001). Among the fungal genera, *Saccharomyces* (enriched in cirrhotic patients) significantly negatively correlated with the PLT index (correlation coefficient < -0.4, *P* < 0.001). *Auricularia* (enriched in healthy individuals) significantly correlated not only with ALB, HGB, and PTA indexes (correlation coefficient>0.5, *P* < 0.001) but also with the MELD index (correlation coefficient < -0.5, *P* < 0.001). Among the metabolites, 4-Acetyl-2-methylpyrimidine and diammonium glycyrrhizinate (enriched in cirrhotic patients) significantly negatively correlated with the PTA index (correlation coefficient < -0.5, *P* < 0.001). 5,6-DHET (enriched in healthy individuals) significantly positively correlated with the PTA, ALB, and HGB indexes (correlation coefficient>0.5, *P* < 0.001).

Then we performed the Spearman's correlation network analysis for the bacterial genera (*n* = 83), fungal genera (*n* = 116), and enriched metabolites (*n* = 68) in 45 cirrhotic patients (LC group). The results showed significant associations (|correlation coefficient|>0.4, *P* < 0.05, FDR corrected) between 74 bacterial genera, 59 fungal genera, and 53 enriched metabolites (Supplementary Figure 5A). Overall, there were 519 positive and 149 negative correlations between different features. Compared to fungi, there are more links between bacteria and enriched metabolites. We also found 3 LC-enriched metabolites were associated with bacterial and fungal genera, but the associations differed (Supplementary Table 1). Within bacteria, 51 genera were associated with each other, of which 10 were enriched in cirrhotic patients (LDA score>3), including *Streptococcus, Ligilactobacillus*, and *Enterococcus*. The top three bacterial genera ranked by degree were *Oscillibacter, Anaerobutyricum* and *Coprococcus*. Within fungi, 46 genera were associated with each other, with *Pichia* enriched in cirrhotic patients (LDA score>3). The top three fungal genera ranked by degree were *Papiliotrema, Absidia*, and *Aureobasidium*. Within metabolites, associations existed among 36 enriched metabolites. The top three metabolites ranked by degree were 7-Methylxanthine, L-Ribulose, and Theophylline. For contrast, we also performed the Spearman's correlation network analysis for the bacterial genera (*n* = 83), fungal genera (n = 116), and enriched metabolites (*n* = 62) in 30 healthy individuals (C group). The results showed significant associations (|correlation coefficient|>0.4, *P* < 0.05, FDR corrected) between 48 bacterial genera, 77 fungal genera, and 46 enriched metabolites (Supplementary Figure 5B). Overall, there were 301 positive and 32 negative correlations between different features.

**Figure 6:**
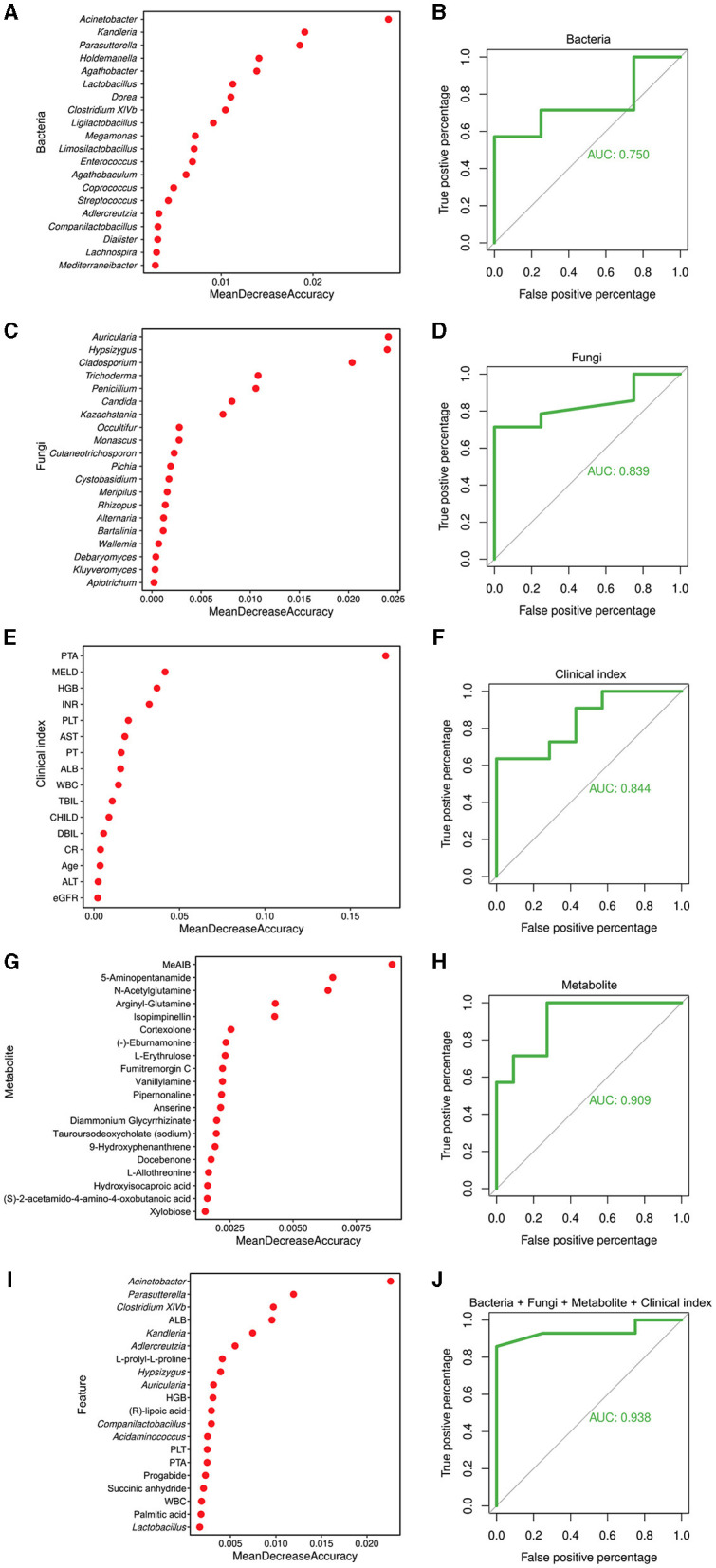
Random forest model for the diagnosis of cirrhosis. **(A)** Top 20 bacterial genera contributing most to the accuracy of predicting sample grouping. **(B)** Bacterial genera used to discriminate between cirrhotic patients and healthy individuals with an AUC of 0.750. **(C)** Top 20 fungal genera contributing most to the accuracy of predicting sample grouping. **(D)** Fungal genera used to discriminate between cirrhotic patients and healthy individuals with an AUC of 0.839. **(E)** Top 20 clinical indexes contributing most to the accuracy of predicting sample grouping. **(F)** Clinical indexes used to discriminate between cirrhotic patients and healthy individuals with an AUC of 0.844. **(G)** Top 20 metabolites contributing most to the accuracy of predicting sample grouping. **(H)** Metabolites used to discriminate between cirrhotic patients and healthy individuals with an AUC of 0.909. **(I)** Top 20 features contributing most to the accuracy of predicting sample grouping. **(J)** Bacterial and fungal genera, metabolites, and clinical indexes used to discriminate between cirrhotic patients and healthy individuals with an AUC of 0.938.

To explore the potential of different biomarkers for diagnosing liver cirrhosis disease, 45 liver cirrhotic patients and 30 healthy individuals were divided into a training set (34LC and 23C) and a test set (11LC and 7C) to construct random forest models based on the 16S data, ITS data, metabolites data, clinical indexes, and a combination of them, respectively. Figures 6A, C, E, G, I showed the top 20 features of different data types that contributed most to the accuracy of predicted sample grouping. The random forest model constructed based on 16S data had the lowest AUC value of 0.75 (Figure 6B). The genera *Acinetobacter* (enriched in LC patients), *Kandleria*, and *Parasutterella* (enriched in healthy individuals) were three of the most important features to this model. Models constructed based on ITS data or clinical indexes had close AUC values of 0.839 and 0.844, respectively (Figures 6D, F). The fungal genera *Auricularia, Hypsizygus*, and *Cladosporium* (enriched in healthy individuals), as well as the clinical indexes PTA, MELD, and HGB were key features. The model constructed on the basis of metabolites data had a high AUC value of 0.909 (Figure 6H). Metabolites MeAIB, 5-Aminopentanamide, and N-Acetylglutamine enriched in LC patients were the top three important features. Compared to other models, the random forest model constructed by combining all four types of features had the highest AUC value of 0.938 (Figure 6J).

## Discussion

It has been widely known that gut microbial dysbiosis is a bidirectional process with the development of cirrhosis because of pathological interactions of the gut-liver axis (Albillos et al., 2020). When the equilibrium between the intestinal microbiome and the microenvironment is disturbed, increased microbial translocation and dysbiosis result in liver injury and are closely linked to the progression of cirrhosis (Acharya et al., 2017). Meanwhile, abnormal liver function and portal hypertension easily occur in the progression of cirrhosis, which will result in intestinal mucosal barrier damage and weakening of the inhibitory effect of the liver on intestinal harmful microbes (Wiest et al., 2014; Acharya and Bajaj, 2017). The intestinal fungi and bacteria share micro-habitats and complexly interact with microbial communities, which have been shown to affect various biological processes maintaining the stability of the intestinal environment (Huang X. et al., 2023). Bacterial and fungal dysbiosis threaten the delicate equilibrium that distinguishes cirrhosis and is associated with a high mortality risk in patients with advanced liver disease through the gut-liver axis (Maraolo et al., 2020; Chen L. et al., 2022). For these reasons, our study focused on the interconnections among cirrhosis-associated gut bacteria, gut fungi, microbial metabolites, and disease conditions.

Previous studies have reported that cirrhotic patients exhibited frequent gastrointestinal bacteria disturbances, which are characterized by a decreased diversity, overgrowth of *Enterococcus*, Enterobacteriaceae, and Bacteroidaceae, and a reduction of the abundance of probiotic microorganisms, such as Lachnospiraceae and Ruminococaceae (Gómez-Hurtado et al., 2016; Wang et al., 2020). Our results in the present study showed that intestinal bacterial alpha diversity was lower than healthy controls (Figure 1A) and identified several compositional bacterial differences in cirrhotic patients. At the genus level, the LC group's relative abundance of pathogenic bacteria such as *Acinetobacter, Enterococcus* and *Streptococcus* significantly increased (Figure 1E), which may cause an inflammatory reaction to aggravate liver injury. It has been reported that *Enterococcus* and *Streptococcus* are significantly abundant in patients with liver diseases, such as autoimmune hepatitis (Ponziani et al., 2018; Zhong et al., 2021; Huang X. Y. et al., 2023). Meanwhile, we observed decreased beneficial bacterium abundance of *Anaerobutyricum, Blautia, Coprococcus, Dorea, Gemmiger*, and *Ruminococcus* in LC group (Figure 1E). KEGG analysis suggested that the variations in gut bacteria led to significant differences in metabolic pathways (Supplementary Figure 1D). In the LC group, more bacteria were enriched in “fatty acid elongation—saturated,” “(5Z)-dodec-5-enoate biosynthesis,” and “inosine-5**′**-phosphate biosynthesis III” pathways. Notably, *Blautia, Coprococcus*, and *Ruminococcus* were mainly involved in producing short-chain fatty acids, which are widely thought to be beneficial microbial substances correlated with a reduced risk of liver fibrosis and cirrhosis (Zhou et al., 2019). Moreover, our study also exhibited strong linkages between gut bacteria and cirrhosis clinical features (Figure 5A), especially the severity of LC, including the MELD score, CTP score, and PTA positively associated with potentially pathogenic taxa and inverse correlations with the probiotic ones.

Analogous to intestinal bacteria disturbances, cirrhotic patients exhibited intestinal fungi disorders with decreased fungal diversity (Figure 2A) and a relative increase in abundance of opportunistic fungal pathogens. In our study, Ascomycota and Basidiomycota were the most common intestinal fungi at the phyla level in LC patients and healthy controls (Figure 2C). Compared with healthy controls, LC patients had an increased abundance of Ascomycota and a decreased abundance of Basidiomycota (Figures 2C, E). As reported, the Basidiomycota to Ascomycota ratio was used to define fungal dysbiosis, and the gut fungal/bacterial metric (Ascomycota/Bacteroidetes) was independently associated with the risk of hospitalization (Bajaj et al., 2018). At the genus level, our results found an increment of *Saccharomyces* and a reduction of *Aspergillus*, and *Penicillium* associated with the clinical variables (Figures 2E, 5B). *Aspergillus, Cryptococcus, Debaryomyces, Malassezia, Penicillium*, and *Saccharomyces* have been more vigorously studied in recent years to highlight the relationship between gut fungi and human diseases, including liver diseases (Suhr and Hallen-Adams, 2015; Doron et al., 2023). Of them, some species of *Saccharomyces* were significantly associated with the severity of the disease in cirrhotic patients (Costa et al., 2014). The increased fecal abundance of *Saccharomyces* was also validated in a cohort of patients with chronic hepatitis B virus (HBV) infection and HBV-associated cirrhosis (Mou et al., 2018). Zhang et al. (2023) have confirmed the decreased alpha diversity and increased *Saccharomycetes* in LC patients with various etiologies. Intriguingly, some edible mushroom genera from dietary sources such as Auricularia and Hypsizygus were found in negatively related to the severity of the liver cirrhosis in our data. Edible mushrooms are healthy food with great therapeutic value as they contain abundant bioactive metabolites with antioxidant, anti-aging, antibacterial, anti-inflammatory properties. When exploring the role of gut fungi in diseases, in addition to colonizing fungi in the intestine itself, fungi from dietary sources may also have a certain effect. In summary, our study demonstrated that intestinal microbial disorders of patients with LC exhibited the lack of potentially beneficial microbes, including an overgrowth of potentially pathogenic microbes (bacteria and fungi), which may affect and reflect the clinical features and severity of liver cirrhosis.

An essential function of the gut microbiota is metabolism, which supplies nutrients to both microbes and host. Recently, increasing evidence has shown that microbial metabolites play pivotal roles in communicating among gut bacteria, fungi, and hosts in various ways, leading to co-occurrence patterns of fungi and bacteria (Gao et al., 2021). Microbial dysbiosis can result in aberrant translocation of microbial components or microbial-derived metabolites (such as bile acids, fatty acids, and tryptophan metabolites) from the gut to the liver via the portal circulation, which can affect the onset and progression of LC (Trebicka et al., 2020). Consistent with the results of intestinal microbial sequencing, we observed the raised prevalence of the “fatty acid elongation—saturated” pathway and the reduced relative abundance of unsaturated and short-chain fatty acid-producing bacteria in patients with LC, suggesting a loss of beneficial fatty acid in the intestinal environment resulting in more pervasive gastrointestinal phenotypes. These metabolites may be involved in regulating bacterial and fungal homeostasis and play an essential role in the development and progression of cirrhosis. This also suggests that metabolites in the gut are probably not just from the same species but may be regulated by multiple ones.

Esophageal and gastric varices is one of the potentially life-threatening complications of LC, resulting in high mortality (Lesmana et al., 2020). Esophageal and gastric variceal bleeding (EGVB) can represent a risk factor for the development of bacterial infections in up to 45% of patients (Tandon and Garcia-Tsao, 2008). In our study, we exhibited the gut microbial ecology in LC patients with EGV. Endoscopic procedures (endoscopic variceal ligation, endoscopic injection sclerotherapy, and endoscopic tissue adhesive) remain the mainstay recommended by domestic and international guidelines for treating EGV and controlling acute bleeding. Changes caused by the endoscopic treatment in portal vein pressure, blood flow, and gastric function may alter gut microbiota composition and abundance. A total of 15 LC patients with EGV who underwent endoscopic treatment were enrolled to investigate the potential roles of endoscopic treatment on gut microbial changes. Finally, no significant differences were found in diversity of gut bacteria and fungi (Figures 4A–D), composition at the phylum level (Supplementary Figures 4B, F), and microbial metabolic pathways before and after endoscopic procedures. However, several bacterial and fungal genera were differentiated after endoscopic treatment (Supplementary Figures 4D, H), which may affect subsequent EGV bleeding. However, it will need long-term follow-up and monitoring.

The gut bacteria and fungi contribute to the intestinal ecosystem through their key roles in host interactions and homeostasis. Fungal–bacterial interactions underlie both the pathogenesis and the progression of disease (Krüger et al., 2019). Previous study found that gut fungi can produce antimicrobial peptides to influence bacterial colonization (Kombrink et al., 2019), while bacteria can modulate fungal commensalism, pathogenesis and growth by affecting fatty acids generation (García et al., 2017; McCrory et al., 2024). The most widely studied pathogenic fungi being antagonized by commensal bacteria (such as *Streptococcus* and *Clostridium difficile*) is *C. albicans* (Santus et al., 2021). Except that, data on the interactions between other fungi and bacteria are limited. Our study preliminarily explored a cirrhosis-specific interkingdom network among bacterial genera, fungal genera, and enriched metabolites (Supplementary Figure 5, Supplementary Table 1), and the type of interactions between these bacteria and fungi might have clinical relevance. Three cirrhosis-enriched metabolites were associated with bacterial and fungal genera, but the associations differed (Supplementary Table 1). Although the roles of these interaction are still unclear, these findings can broaden our knowledge of the function and crosstalk of gut microbiota.

Gut microbes have been used as novel molecular biomarkers, mainly for disease risk prediction modeling, and have proved their value in several diseases (e.g., diabetes, enteritis, and bowel cancer). Previous studied have interrogated shotgun metagenomic, untargeted metabolomic profiles and random forest machine learning algorithm to evaluate the diagnostic accuracy based on a set of liver fibrosis/cirrhosis-related bacterial and metabolomic signatures, and the result were effective (AUC: 0.72–0.91; Loomba et al., 2017; Lee et al., 2020; Oh et al., 2020). The construction of disease risk models for liver cirrhosis was also attempted in our study with multiple data combinations. Our modeling was based on gut microbiome data and clinical indexes from 45 patients with cirrhosis and 30 healthy individuals, with AUCs ranging from 0.750 to 0.938. Moreover, we found that the efficacy of diagnostic models based on fungal signatures is not inferior to those based on bacterial or metabolic signatures. Combining the metagenomic signature with key clinical indicators (PTA, MELD, and HGB) accurately distinguished cirrhosis in etiologically can improve the diagnostic efficacy of liver cirrhosis.

Of course, several issues should also be addressed. Firstly, we used an insufficient number of samples (case: 45 vs. control:30), thus we did not classify cirrhosis patients in detail, and differences in disease subtypes, which has an impact on our screening of differential bacteria, fungi, and metabolites. Secondly, the overall performance of our models was not promising enough, there is still much room for improvement in the AUC of the risk prediction model as well as its stability. Thirdly, this study mainly used amplicon sequencing to study bacteria and fungi in the gut microbiota. Therefore, more large-scale research must be done on the genes and functions of bacteria and fungi by mNGS sequencing. We plan to remedy these deficiencies in subsequent studies.

## Conclusion

In conclusion, our study presented the cirrhosis-associated intestinal microbial characteristics and highlighted the gut bacteria-fungi-metabolite interactions in patients with cirrhosis. Meanwhile, we constructed a predicted diagnosis of cirrhosis using a random forest model based on identified intestinal microbial and clinical features. This interaction provides a new direction and theoretical basis for exploring gut microbiota-based diagnostic and therapeutic strategies for cirrhotic patients. Further mechanistic studies of fungal, bacterial, and metabolic ecological interactions in liver cirrhosis are required.

## Data Availability

The datasets presented in this study can be found in online repositories. The names of the repository/repositories and accession number(s) can be found at: https://www.ncbi.nlm.nih.gov/, PRJNA1028775.

## Supplementary material

Supplementary Figure 1 Analysis of intestinal bacterial communities in cirrhotic patients. **(A)** Rarefaction curves. **(B)** Presence of ASVs (relative abundance > 0.1%) within different subgroups. **(C)** PCA analysis of the functional composition of communities in different subgroups. **(D)** Differential pathways in different subgroups (difference between proportions > 0.17%, *q*-value < 0.01). Pathway names not shown in full are adenosylcobalamin biosynthesis from cobyrinate a,c-diamide I, superpathway of guanosine nucleotides *de novo* biosynthesis I, superpathway of guanosine nucleotides *de novo* biosynthesis II.

Supplementary Figure 2 Analysis of intestinal fungal communities in cirrhotic patients. **(A)** Rarefaction curves. **(B)** Presence of ASVs (relative abundance > 0.1%) within different subgroups. **(C)** PCA analysis of the functional composition of communities in different subgroups. **(D)** Differential pathways (difference between proportions > 0.3%, *q*-value < 0.01) in different subgroups. Pathway names not shown in full are NAD/NADP-NADH/NADPH mitochondrial interconversion (yeast), phospholipid remodeling (phosphatidylethanolamine, yeast).

Supplementary Figure 3 Analysis of intestinal metabolites in cirrhotic patients. **(A)** Distribution of metabolites differing between groups. Metabolites enriched in group C are shown in blue, metabolites enriched in group LC are shown in red, and metabolites without significant differences are shown in gray. **(B)** Distribution of the 130 identified differential metabolites among samples. Darker colors indicate higher relative abundance of metabolites. Red color represents 45 patients with liver cirrhosis (LC group) and blue color represents 30 healthy control individuals (C group). **(C)** Results of KEGG pathway enrichment analysis of differential metabolites (Top 30). Vertical coordinates are KEGG pathways enriched for differential metabolites. The horizontal coordinate, Rich factor, is the ratio of the number of differential metabolites in the corresponding pathway to the total number of metabolites detected and annotated in that pathway. A larger value indicates a greater degree of enrichment. The size of the dot represents the number of differential metabolites enriched. The color of the dots is the hypergeometric test *p*-value, with redder values indicating more significant enrichment.

Supplementary Figure 4 Analysis of intestinal bacterial and fungal communities and metabolites in patients with liver cirrhosis after the endoscopic treatment. **(A)** Rarefaction curves (bacteria). **(B)** Bacterial community composition (phylum level). **(C)** Bacterial community composition (genus level). **(D)** LEfSe analysis reveals differential bacteria between groups (LDA score > 3). **(E)** Rarefaction curves (fungi). **(F)** Fungal community composition (phylum level). **(G)** Fungal community composition (genus level). **(H)** LEfSe analysis reveals differential fungi between groups (LDA score > 3). **(I)** Distribution of differential metabolites in cirrhotic patients before and after treatment. Red color represents metabolites enriched in TA group and blue color represents metabolites enriched in TB group. **(J)** Distribution of 16 identified differential metabolites in samples between groups. **(K)** Results of KEGG pathway enrichment analysis of 16 identified differential metabolites.

Supplementary Figure 5 Correlation network of enriched metabolites with bacteria and fungi. **(A)** LC group network. **(B)** C group network. Blue triangles, red squares and cyan circles represent bacterial genera, fungal genera, and LC-enriched metabolites, respectively. Larger nodes have more associations with other nodes. Red lines represent positive correlations and blue lines represent negative correlations. Spearman's correlation coefficient|>0.4, *P* < 0.05 (FDR corrected).

## References

[B1] Abarenkov K. Nilsson R. H. Larsson K.-H. Taylor A. F. S. May T. W. Frøslev T. G.. (2023). The UNITE database for molecular identification and taxonomic communication of fungi and other eukaryotes: sequences, taxa and classifications reconsidered. Nucl. Acids Res. 2023:gkad1039. 10.1093/nar/gkad1039 37953409 PMC10767974

[B2] Acharya C. Bajaj J. S. (2017). Gut microbiota and complications of liver disease. Gastroenterol. Clin. N. Am. 46, 155–169. 10.1016/j.gtc.2016.09.013 28164848 PMC5300079

[B3] Acharya C. Sahingur S. E. Bajaj J. S. (2017). Microbiota, cirrhosis, and the emerging oral-gut-liver axis. JCI Insight 2:94416. 10.1172/jci.insight.94416 28978799 PMC5841881

[B4] Albillos A. de Gottardi A. Rescigno M. (2020). The gut-liver axis in liver disease: pathophysiological basis for therapy. J. Hepatol. 72, 558–577. 10.1016/j.jhep.2019.10.003 31622696

[B5] Bajaj J. S. Heuman D. M. Hylemon P. B. Sanyal A. J. White M. B. Monteith P.. (2014). Altered profile of human gut microbiome is associated with cirrhosis and its complications. J. Hepatol. 60, 940–947. 10.1016/j.jhep.2013.12.019 24374295 PMC3995845

[B6] Bajaj J. S. Liu E. J. Kheradman R. Fagan A. Heuman D. M. White M.. (2018). Fungal dysbiosis in cirrhosis. Gut 67, 1146–1154. 10.1136/gutjnl-2016-313170 28578302

[B7] Caspi R. Billington R. Fulcher C. A. Keseler I. M. Kothari A. Krummenacker M.. (2018). The MetaCyc database of metabolic pathways and enzymes. Nucl. Acids Res. 46, D633–D639. 10.1093/nar/gkx935 29059334 PMC5753197

[B8] Chen L. Zhu Y. Hou X. Yang L. Chu H. (2022). The role of gut bacteria and fungi in alcohol-associated liver disease. Front. Med. 9:840752. 10.3389/fmed.2022.840752 35308525 PMC8927088

[B9] Chen T. Liu Y. X. Huang L. (2022). ImageGP: an easy-to-use data visualization web server for scientific researchers. iMeta 1:5. 10.1002/imt2.5 38867732 PMC10989750

[B10] Cole J. R. Wang Q. Fish J. A. Chai B. McGarrell D. M. Sun Y.. (2014). Ribosomal Database Project: data and tools for high throughput rRNA analysis. Nucl. Acids Res. 42, D633–D642. 10.1093/nar/gkt1244 24288368 PMC3965039

[B11] Costa L. J. Abreu F. M. d. L. Garcia V. E. Soares L. A. Davisson C. M. I. T. (2014). The effect of *Saccharomyces boulardii* in patients eligible for liver transplantation. Nutricion Hospitalaria 31, 778–784. 10.3305/nh.2015.31.2.7949 25617563

[B12] Doron I. Kusakabe T. Iliev I. D. (2023). Immunoglobulins at the interface of the gut mycobiota and anti-fungal immunity. Semin. Immunol. 67:101757. 10.1016/j.smim.2023.101757 37003056 PMC10192079

[B13] Douglas G. M. Maffei V. J. Zaneveld J. R. Yurgel S. N. Brown J. R. Taylor C. M.. (2020). PICRUSt2 for prediction of metagenome functions. Nat. Biotechnol. 38, 685–688. 10.1038/s41587-020-0548-6 32483366 PMC7365738

[B14] Dworecka-Kaszak B. Dabrowska I. Kaszak I. (2016). The mycobiome—a friendly cross-talk between fungal colonizers and their host. Ann. Parasitol. 62, 175–184. 27770757 10.17420/ap6203.51

[B15] Edgar R. C. (2010). Search and clustering orders of magnitude faster than BLAST. Bioinformatics 26, 2460–2461. 10.1093/bioinformatics/btq461 20709691

[B16] Gao B. Zhang X. Schnabl B. (2021). Fungi-bacteria correlation in alcoholic hepatitis patients. Toxins 13:20143. 10.3390/toxins13020143 33672887 PMC7917833

[B17] Gao Y. Zhang G. Jiang S. Liu Y. X. (2024). Wekemo bioincloud: a user-friendly platform for meta-omics data analyses. Imeta 3:e175. 10.1002/imt2.175 38868508 PMC10989175

[B18] García C. Tebbji F. Daigneault M. Liu N.-N. Köhler J. R. Allen-Vercoe E.. (2017). The human gut microbial metabolome modulates fungal growth via the TOR signaling pathway. mSphere 2:17. 10.1128/mSphere.00555-17 29242837 PMC5729221

[B19] Gomaa E. Z. (2020). Human gut microbiota/microbiome in health and diseases: a review. Antonie Van Leeuwenhoek 113, 2019–2040. 10.1007/s10482-020-01474-7 33136284

[B20] Gómez-Hurtado I. Such J. Francés R. (2016). Microbiome and bacterial translocation in cirrhosis. Gastroenterología y Hepatología 39, 687–696. 10.1016/j.gastrohep.2015.10.013 26775042

[B21] Hsu C. L. Schnabl B. (2023). The gut-liver axis and gut microbiota in health and liver disease. Nat. Rev. Microbiol. 21, 719–733. 10.1038/s41579-023-00904-3 37316582 PMC10794111

[B22] Huang X. Hu M. Sun T. Li J. Zhou Y. Yan Y.. (2023). Multi-kingdom gut microbiota analyses define bacterial-fungal interplay and microbial markers of pan-cancer immunotherapy across cohorts. Cell Host Microbe 31, 1930–1943.e1934. 10.1016/j.chom.2023.10.005 37944495

[B23] Huang X. Y. Zhang Y.-H. Yi S.-Y. Lei L. Ma T. Huang R.. (2023). Potential contribution of the gut microbiota to the development of portal vein thrombosis in liver cirrhosis. Front. Microbiol. 14:1217338. 10.3389/fmicb.2023.1217338 37965548 PMC10641681

[B24] Kanehisa M. (2000). KEGG: kyoto encyclopedia of genes and genomes. Nucl. Acids Res. 28, 27–30. 10.1093/nar/28.1.27 10592173 PMC102409

[B25] Kisseleva T. Brenner D. (2020). Molecular and cellular mechanisms of liver fibrosis and its regression. Nat. Rev. Gastroenterol. Hepatol. 18, 151–166. 10.1038/s41575-020-00372-7 33128017

[B26] Kombrink A. Tayyrov A. Essig A. Stöckli M. Micheller S. Hintze J.. (2019). Induction of antibacterial proteins and peptides in the coprophilous mushroom *Coprinopsis cinerea* in response to bacteria. ISME J. 13, 588–602. 10.1038/s41396-018-0293-8 30301946 PMC6461984

[B27] Krüger W. Vielreicher S. Kapitan M. Jacobsen I. D. Niemiec M. J. (2019). Fungal-bacterial interactions in health and disease. Pathogens 8:20070. 10.3390/pathogens8020070 31117285 PMC6630686

[B28] Lachar J. Bajaj J. (2016). Changes in the microbiome in cirrhosis and relationship to complications: hepatic encephalopathy, spontaneous bacterial peritonitis, and sepsis. Semin. Liv. Dis. 36, 327–330. 10.1055/s-0036-1593881 27997972

[B29] Lee G. You H. J. Bajaj J. S. Joo S. K. Yu J. Park S.. (2020). Distinct signatures of gut microbiome and metabolites associated with significant fibrosis in non-obese NAFLD. Nat. Commun. 11:4982. 10.1038/s41467-020-18754-5 33020474 PMC7536225

[B30] Lesmana C. R. A. Raharjo M. Gani R. A. (2020). Managing liver cirrhotic complications: overview of esophageal and gastric varices. Clin. Mol. Hepatol. 26, 444–460. 10.3350/cmh.2020.0022 33053928 PMC7641566

[B31] Liu Y.-X. Chen L. Ma T. Li X. Zheng M. Zhou X.. (2023). EasyAmplicon: an easy-to-use, open-source, reproducible, and community-based pipeline for amplicon data analysis in microbiome research. iMeta 2:83. 10.1002/imt2.83 38868346 PMC10989771

[B32] Llorente C. Schnabl B. (2015). The gut microbiota and liver disease. Cell. Mol. Gastroenterol. Hepatol. 1, 275–284. 10.1016/j.jcmgh.2015.04.003 26090511 PMC4467911

[B33] Loomba R. Seguritan V. Li W. Long T. Klitgord N. Bhatt A.. (2017). Gut microbiome-based metagenomic signature for non-invasive detection of advanced fibrosis in human nonalcoholic fatty liver disease. Cell Metab. 25, 1054–1062.e1055. 10.1016/j.cmet.2017.04.001 28467925 PMC5502730

[B34] Lynch S. V. Phimister E. G. Pedersen O. (2016). The human intestinal microbiome in health and disease. N. Engl. J. Med. 375, 2369–2379. 10.1056/NEJMra1600266 27974040

[B35] Maraolo A. E. Scotto R. Zappulo E. Pinchera B. Moriello N. S. Nappa S.. (2020). Novel strategies for the management of bacterial and fungal infections in patients with liver cirrhosis: focus on new antimicrobials. Expert Rev. Anti-Infect. Ther. 18, 191–202. 10.1080/14787210.2020.1725473 32011191

[B36] McCrory C. Lenardon M. Traven A. (2024). Bacteria-derived short-chain fatty acids as potential regulators of fungal commensalism and pathogenesis. Trends Microbiol. 4:4. 10.1016/j.tim.2024.04.004 38729839

[B37] Moon A. M. Singal A. G. Tapper E. B. (2020). Contemporary epidemiology of chronic liver disease and cirrhosis. Clin. Gastroenterol. Hepatol. 18, 2650–2666. 10.1016/j.cgh.2019.07.060 31401364 PMC7007353

[B38] Mou H. Yang F. Zhou J. Bao C. (2018). Correlation of liver function with intestinal flora, vitamin deficiency and IL-17A in patients with liver cirrhosis. Exp. Therapeut. Med. 2018:6663. 10.3892/etm.2018.6663 30344685 PMC6176138

[B39] Oh T. G. Kim S. M. Caussy C. Fu T. Guo J. Bassirian S.. (2020). A universal gut-microbiome-derived signature predicts cirrhosis. Cell Metab 32, 878–888.e876. 10.1016/j.cmet.2020.06.005 32610095 PMC7822714

[B40] Pabst O. Hornef M. W. Schaap F. G. Cerovic V. Clavel T. Bruns T. (2023). Gut-liver axis: barriers and functional circuits. Nat. Rev. Gastroenterol. Hepatol. 20, 447–461. 10.1038/s41575-023-00771-6 37085614

[B41] Parks D. H. Tyson G. W. Hugenholtz P. Beiko R. G. (2014). STAMP: statistical analysis of taxonomic and functional profiles. Bioinformatics 30, 3123–3124. 10.1093/bioinformatics/btu494 25061070 PMC4609014

[B42] Ponziani F. R. Bhoori S. Castelli C. Putignani L. Rivoltini L. Del Chierico F.. (2018). Hepatocellular carcinoma is associated with gut microbiota profile and inflammation in nonalcoholic fatty liver disease. Hepatology 69, 107–120. 10.1002/hep.30036 29665135

[B43] Qin N. Yang F. Li A. Prifti E. Chen Y. Shao L.. (2014). Alterations of the human gut microbiome in liver cirrhosis. Nature 513, 59–64. 10.1038/nature13568 25079328

[B44] Quast C. Pruesse E. Yilmaz P. Gerken J. Schweer T. Yarza P.. (2012). The SILVA ribosomal RNA gene database project: improved data processing and web-based tools. Nucl. Acids Res. 41, D590–D596. 10.1093/nar/gks1219 23193283 PMC3531112

[B45] Rognes T. Flouri T. Nichols B. Quince C. Mahé F. (2016). VSEARCH: a versatile open source tool for metagenomics. PeerJ 4:2584. 10.7717/peerj.2584 27781170 PMC5075697

[B46] Santus W. Devlin J. R. Behnsen J. (2021). Crossing kingdoms: how the mycobiota and fungal-bacterial interactions impact host health and disease. Infect Immun. 89:20. 10.1128/IAI.00648-20 33526565 PMC8090948

[B47] Schnabl B. Brenner D. A. (2014). Interactions between the intestinal microbiome and liver diseases. Gastroenterology 146, 1513–1524. 10.1053/j.gastro.2014.01.020 24440671 PMC3996054

[B48] Shao L. Ling Z. Chen D. Liu Y. Yang F. Li L. (2018). Disorganized gut microbiome contributed to liver cirrhosis progression: a meta-omics-based study. Front. Microbiol. 9:3166. 10.3389/fmicb.2018.03166 30631318 PMC6315199

[B49] Suhr M. J. Hallen-Adams H. E. (2015). The human gut mycobiome: pitfalls and potentials–a mycologists perspective. Mycologia 107, 1057–1073. 10.3852/15-147 26354806

[B50] Sun D. L. Jiang X. Wu Q. L. Zhou N. Y. (2013). Intragenomic heterogeneity of 16S rRNA genes causes overestimation of prokaryotic diversity. Appl. Environ. Microbiol. 79, 5962–5969. 10.1128/AEM.01282-13 23872556 PMC3811346

[B51] Tandon P. Garcia-Tsao G. (2008). Bacterial infections, sepsis, and multiorgan failure in cirrhosis. Semin. Liv. Dis. 28, 026–042. 10.1055/s-2008-1040319 18293275

[B52] Theocharidou E. Agarwal B. Jeffrey G. Jalan R. Harrison D. Burroughs A. K.. (2016). Early invasive fungal infections and colonization in patients with cirrhosis admitted to the intensive care unit. Clin. Microbiol. Infect. 22, 189.e181–189.e187. 10.1016/j.cmi.2015.10.020 26551838

[B53] Trebicka J. Bork P. Krag A. Arumugam M. (2020). Utilizing the gut microbiome in decompensated cirrhosis and acute-on-chronic liver failure. Nat. Rev. Gastroenterol. Hepatol. 18, 167–180. 10.1038/s41575-020-00376-3 33257833

[B54] Tripathi A. Debelius J. Brenner D. A. Karin M. Loomba R. Schnabl B.. (2018). The gut-liver axis and the intersection with the microbiome. Nat. Rev. Gastroenterol. Hepatol. 15, 397–411. 10.1038/s41575-018-0011-z 29748586 PMC6319369

[B55] Verma N. Roy A. Singh S. Pradhan P. Garg P. Singh M. (2022). Factors determining the mortality in cirrhosis patients with invasive candidiasis: a systematic review and meta-analysis. Med. Mycol. 60:myab069. 10.1093/mmy/myab069 34734272

[B56] Wang R. Tang R. Li B. Ma X. Schnabl B. Tilg H. (2020). Gut microbiome, liver immunology, and liver diseases. Cell. Mol. Immunol. 18, 4–17. 10.1038/s41423-020-00592-6 33318628 PMC7852541

[B57] Wiest R. Lawson M. Geuking M. (2014). Pathological bacterial translocation in liver cirrhosis. J. Hepatol. 60, 197–209. 10.1016/j.jhep.2013.07.044 23993913

[B58] Yan A. W. Fouts D. E. Brandl J. Stärkel P. Torralba M. Schott E.. (2011). Enteric dysbiosis associated with a mouse model of alcoholic liver disease. Hepatology 53, 96–105. 10.1002/hep.24018 21254165 PMC3059122

[B59] Zeng S. Schnabl B. (2022). Roles for the mycobiome in liver disease. Liver Int. 42, 729–741. 10.1111/liv.15160 34995410 PMC8930708

[B60] Zhang L. Chen C. Chai D. Li C. Qiu Z. Kuang T.. (2023). Characterization of the intestinal fungal microbiome in patients with hepatocellular carcinoma. J. Transl. Med. 21:3940. 10.1186/s12967-023-03940-y 36793057 PMC9933289

[B61] Zhong X. Cui P. Jiang J. Ning C. Liang B. Zhou J.. (2021). Streptococcus, the Predominant Bacterium to Predict the Severity of Liver Injury in Alcoholic Liver Disease. Frontiers in Cellular and Infection Microbiology. 11. 10.3389/fcimb.2021.649060 33816353 PMC8010180

[B62] Zhou D. Fan J.-G. (2019). Microbial metabolites in non-alcoholic fatty liver disease. World Journal of Gastroenterology. 25, 2019–2028. 10.3748/wjg.v25.i17.2019 31114130 PMC6506577

