# Supplementary figures and images for: The signatures and crosstalk of gut microbiome, mycobiome, and metabolites in decompensated cirrhotic patients
Source: Front Microbiol. 2024 Aug 21;15:1443182. doi: 10.3389/fmicb.2024.1443182 (PMC11372394; doi:10.3389/fmicb.2024.1443182)

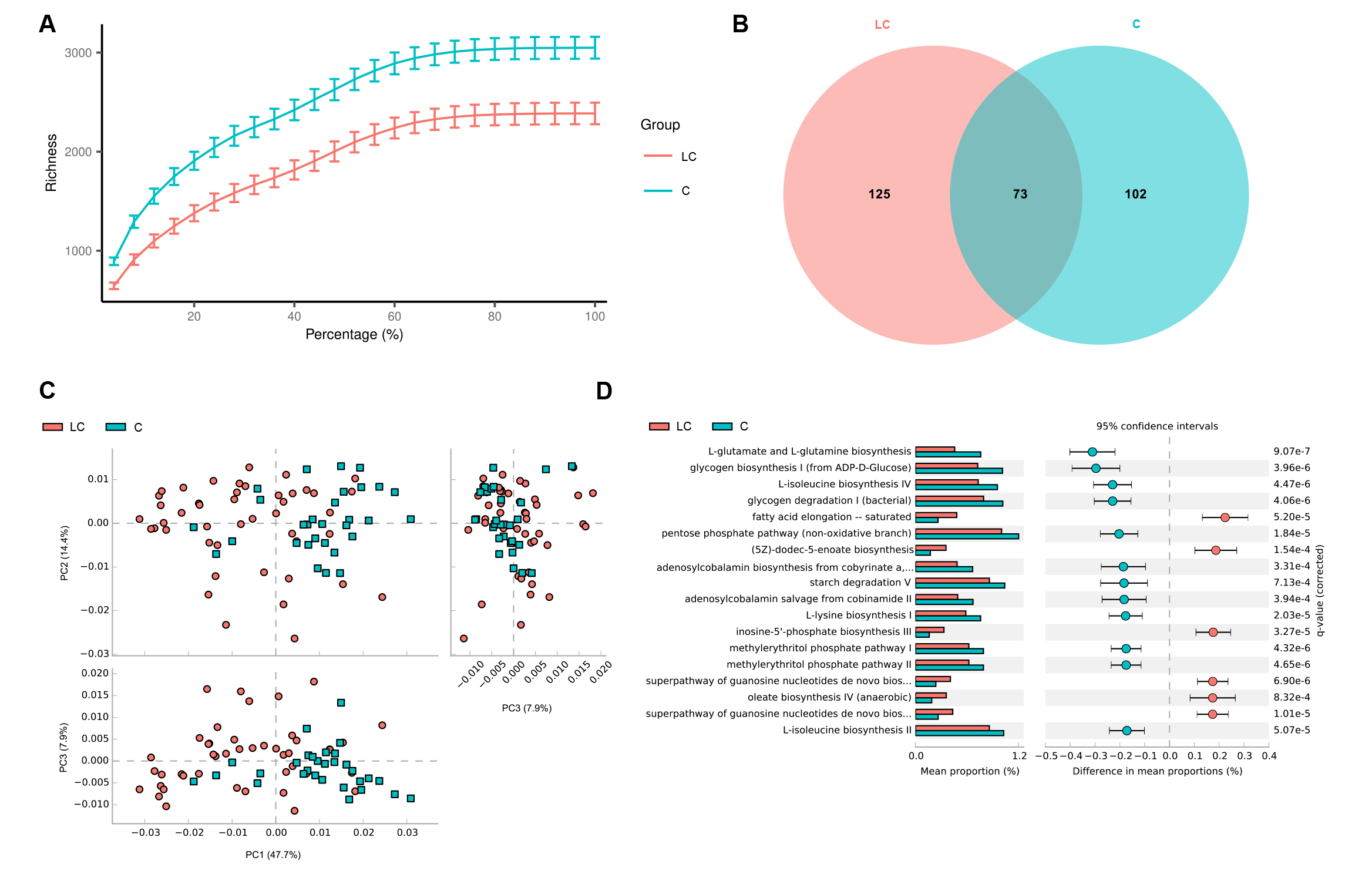

Supplement: Supplementary Figure 1 — Analysis of intestinal bacterial communities in cirrhotic patients. (A) Rarefaction curves. (B) Presence of ASVs (relative abundance > 0.1%) within different subgroups. (C) PCA analysis of the functional composition of communities in different subgroups. (D) Differential pathways in different subgroups (difference between proportions > 0.17%, q-value < 0.01). Pathway names not shown in full are adenosylcobalamin biosynthesis from cobyrinate a,c-diamide I, superpathway of guanosine nucleotides de novo biosynthesis I, superpathway of guanosine nucleotides de novo biosynthesis II. [file Image_1.TIF]

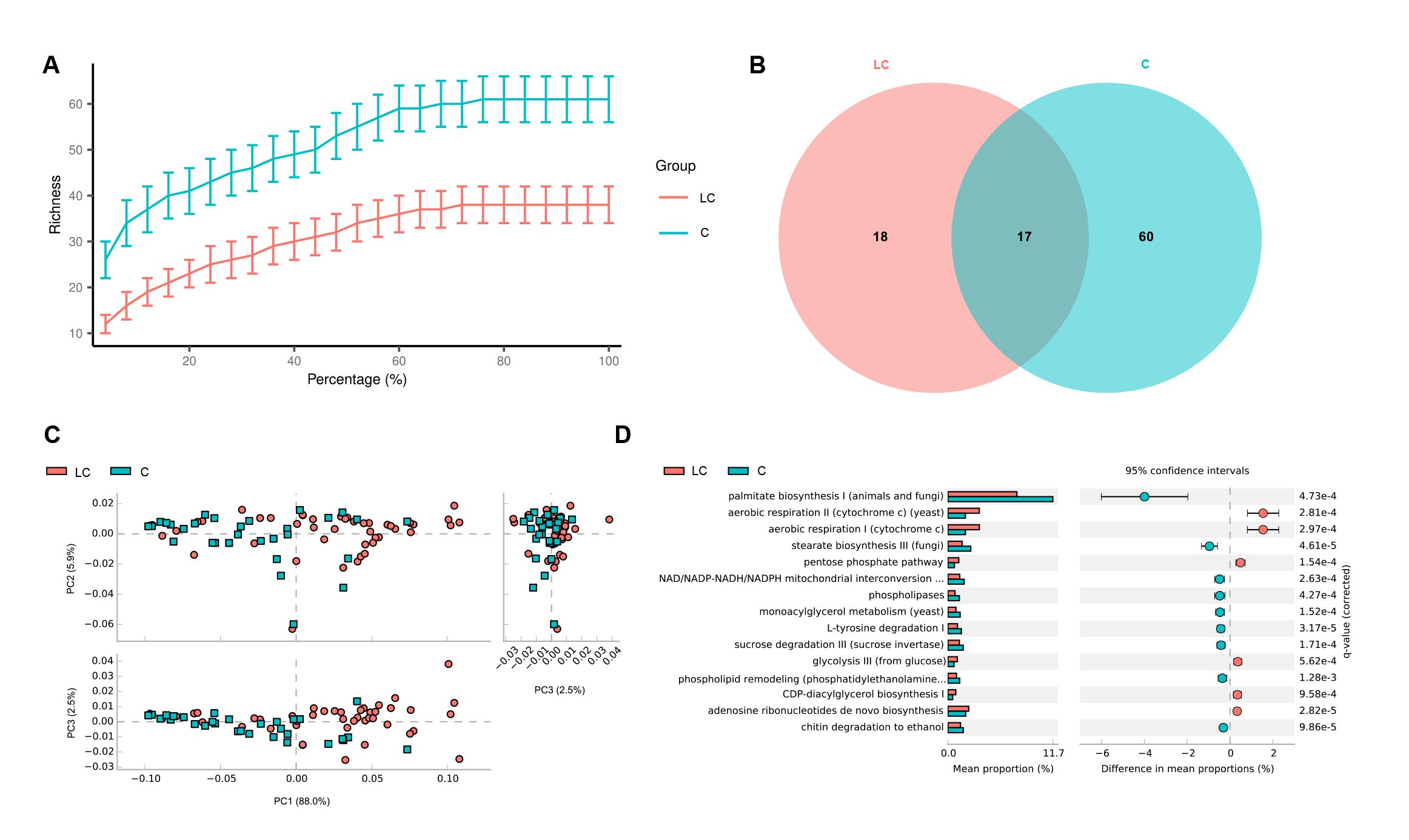

Supplement: Supplementary Figure 2 — Analysis of intestinal fungal communities in cirrhotic patients. (A) Rarefaction curves. (B) Presence of ASVs (relative abundance > 0.1%) within different subgroups. (C) PCA analysis of the functional composition of communities in different subgroups. (D) Differential pathways (difference between proportions > 0.3%, q-value < 0.01) in different subgroups. Pathway names not shown in full are NAD/NADP-NADH/NADPH mitochondrial interconversion (yeast), phospholipid remodeling (phosphatidylethanolamine, yeast). [file Image_2.TIF]

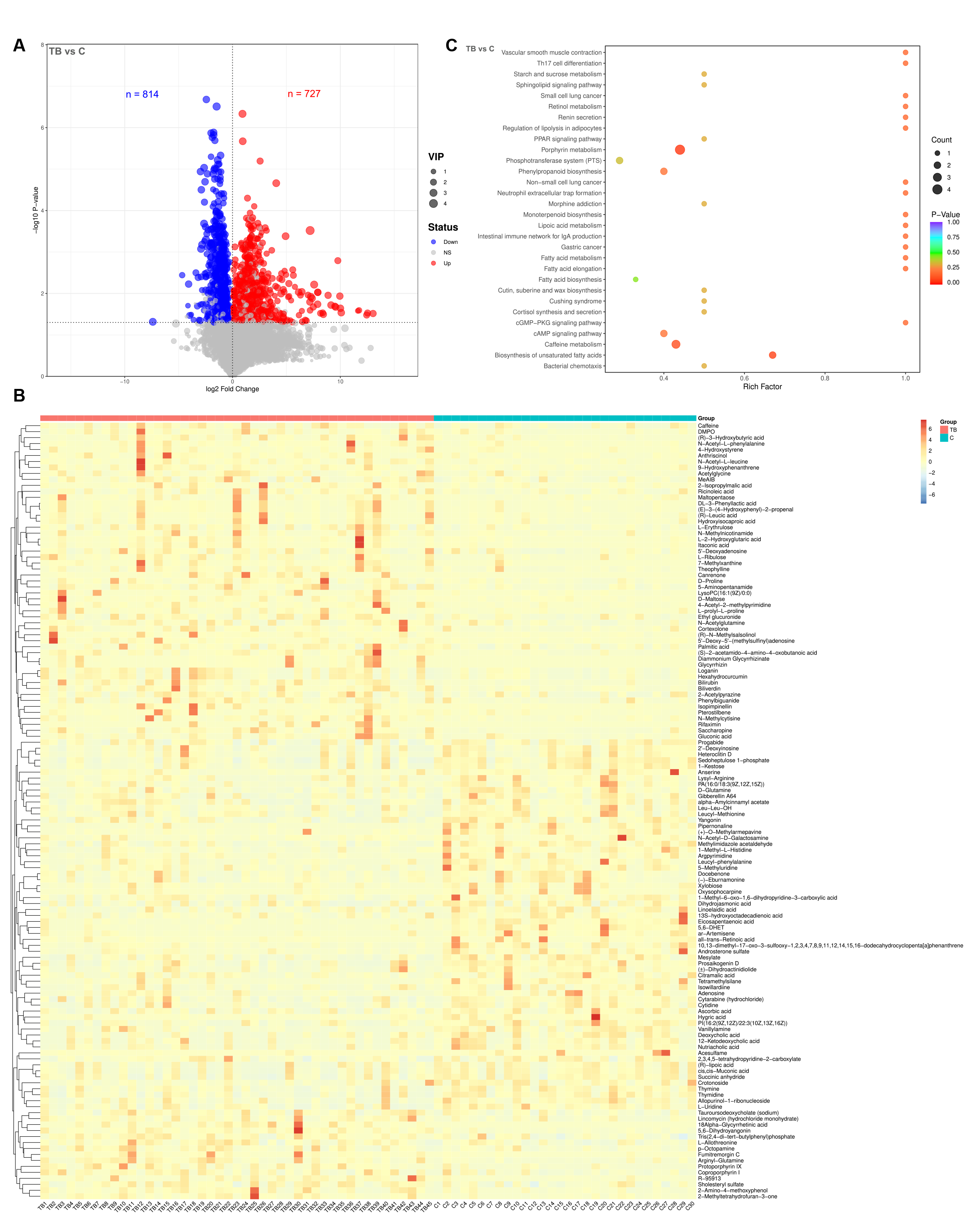

Supplement: Supplementary Figure 3 — Analysis of intestinal metabolites in cirrhotic patients. (A) Distribution of metabolites differing between groups. Metabolites enriched in group C are shown in blue, metabolites enriched in group LC are shown in red, and metabolites without significant differences are shown in gray. (B) Distribution of the 130 identified differential metabolites among samples. Darker colors indicate higher relative abundance of metabolites. Red color represents 45 patients with liver cirrhosis (LC group) and blue color represents 30 healthy control individuals (C group). (C) Results of KEGG pathway enrichment analysis of differential metabolites (Top 30). Vertical coordinates are KEGG pathways enriched for differential metabolites. The horizontal coordinate, Rich factor, is the ratio of the number of differential metabolites in the corresponding pathway to the total number of metabolites detected and annotated in that pathway. A larger value indicates a greater degree of enrichment. The size of the dot represents the number of differential metabolites enriched. The color of the dots is the hypergeometric test p-value, with redder values indicating more significant enrichment. [file Image_3.TIF]

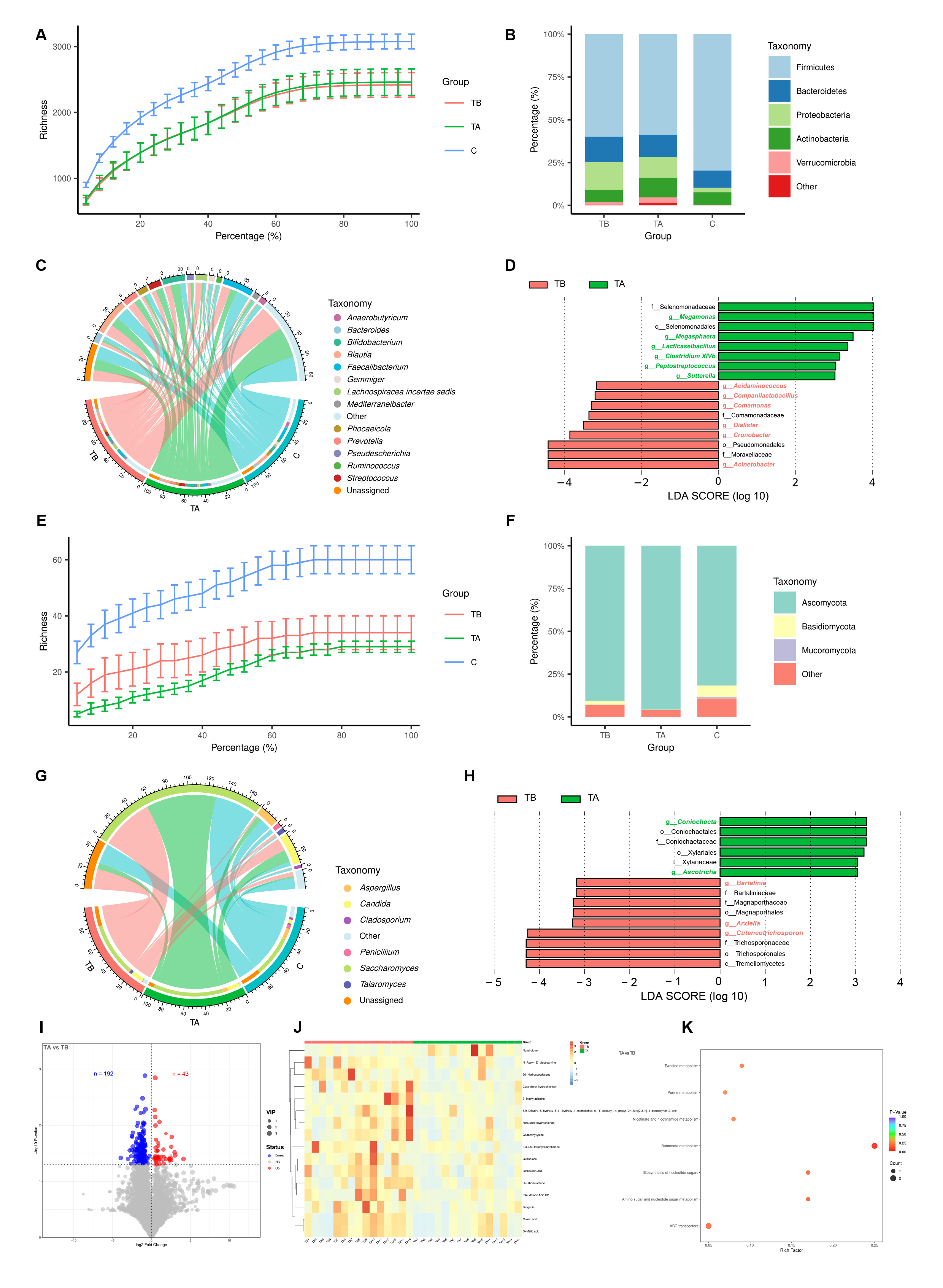

Supplement: Supplementary Figure 4 — Analysis of intestinal bacterial and fungal communities and metabolites in patients with liver cirrhosis after the endoscopic treatment. (A) Rarefaction curves (bacteria). (B) Bacterial community composition (phylum level). (C) Bacterial community composition (genus level). (D) LEfSe analysis reveals differential bacteria between groups (LDA score > 3). (E) Rarefaction curves (fungi). (F) Fungal community composition (phylum level). (G) Fungal community composition (genus level). (H) LEfSe analysis reveals differential fungi between groups (LDA score > 3). (I) Distribution of differential metabolites in cirrhotic patients before and after treatment. Red color represents metabolites enriched in TA group and blue color represents metabolites enriched in TB group. (J) Distribution of 16 identified differential metabolites in samples between groups. (K) Results of KEGG pathway enrichment analysis of 16 identified differential metabolites. [file Image_4.TIF]

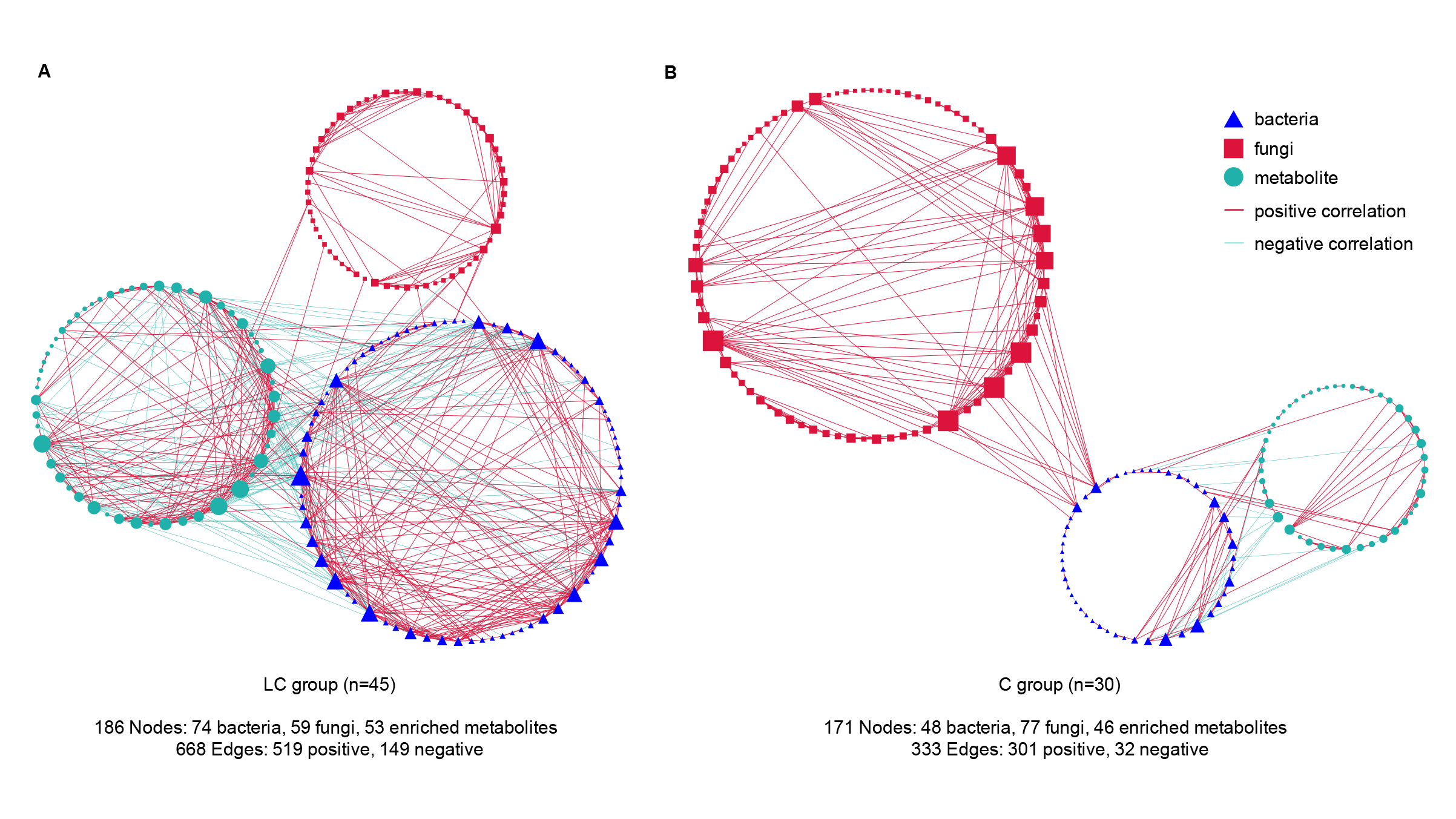

Supplement: Supplementary Figure 5 — Correlation network of enriched metabolites with bacteria and fungi. (A) LC group network. (B) C group network. Blue triangles, red squares and cyan circles represent bacterial genera, fungal genera, and LC-enriched metabolites, respectively. Larger nodes have more associations with other nodes. Red lines represent positive correlations and blue lines represent negative correlations. Spearman's correlation coefficient|>0.4, P < 0.05 (FDR corrected). [file Image_5.TIF]
