# Supplementary material for: The signatures and crosstalk of gut microbiome, mycobiome, and metabolites in decompensated cirrhotic patients
Source: Front Microbiol. 2024 Aug 21;15:1443182. doi: 10.3389/fmicb.2024.1443182 (PMC11372394; doi:10.3389/fmicb.2024.1443182)
Supplement: Supplementary file 6 [file Table_1.DOCX]

Table S1 Correlations between LC group-enriched metabolites and microbial genera

| Genus | Taxonomy | Enriched Metabolite | Correlation Type |
| --- | --- | --- | --- |
| *Prevotella* | Bacteria | Isopimpinellin | positive |
| Veillonella (LC, LDA score > 3) | Bacteria | Anthriscinol | positive |
| Holdemanella (C, LDA score > 3) | Bacteria | Lincomycin (hydrochloride monohydrate) | positive |
| Mediterraneibacter (C, LDA score > 3) | Bacteria | 5-Aminopentanamide | negative |
| Coprococcus (C, LDA score > 3) | Bacteria | N-Acetylglutamine | negative |
| *Lachnospiracea incertae sedis* | Bacteria | Cortexolone | negative |
| *Lachnospiracea incertae sedis* | Bacteria | MeAIB | negative |
| *Lachnospiracea incertae sedis* | Bacteria | 5-Deoxyadenosine | positive |
| *Alistipes* (C, LDA score > 3) | Bacteria | Protoporphyrin IX | positive |
| *Dorea* (C, LDA > 4) | Bacteria | N-Acetylglutamine | negative |
| *Dorea* (C, LDA > 4) | Bacteria | Protoporphyrin IX | positive |
| *Akkermansia* (LC, LDA score > 4) | Bacteria | 5'-Deoxy-5'-(methylsulfinyl)adenosine | positive |
| *Enterocloster* (LC, LDA score > 3) | Bacteria | L-prolyl-L-proline | positive |
| *Erysipelatoclostridium* | Bacteria | 2-Acetylpyrazine | negative |
| *Erysipelatoclostridium* | Bacteria | Protoporphyrin IX | negative |
| *Clostridium XlVb* (C, LDA score > 3) | Bacteria | L-Allothreonine | negative |
| *Clostridium XlVb* (C, LDA score > 3) | Bacteria | Pterostilbene | positive |
| *Adlercreutzia* (C, LDA score > 2) | Bacteria | 5'-Deoxy-5'-(methylsulfinyl)adenosine | positive |
| *Adlercreutzia* (C, LDA score > 2) | Bacteria | 5,6-Dihydroyangonin | negative |
| *Clostridium XVIII* | Bacteria | DMPO | negative |
| *Desulfovibrio* (C, LDA score > 2) | Bacteria | N-Methylnicotinamide | positive |
| *Eggerthella* | Bacteria | Cortexolone | negative |
| *Eggerthella* | Bacteria | MeAIB | negative |
| *Colidextribacter* | Bacteria | Protoporphyrin IX | positive |
| *Aspergillus* (C, LDA score > 4) | Fungi | Protoporphyrin IX | positive |
| *Fusarium* | Fungi | 4-Hydroxystyrene | negative |
| *Talaromyces* | Fungi | L-prolyl-L-proline | positive |
| *Wallemia* (C, LDA score > 3) | Fungi | p-Octopamine | positive |
| *Candida* | Fungi | Cortexolone | positive |
| *Apiotrichum* | Fungi | Protoporphyrin IX | positive |
| *Cyphellophora* | Fungi | L-Ribulose | positive |
| *Debaryomyces*(LC, LDA score > 2) | Fungi | Tauroursodeoxycholate (sodium) | positive |
| *Debaryomyces*(LC, LDA score > 2) | Fungi | Itaconic acid | negative |
| *Debaryomyces*(LC, LDA score > 2) | Fungi | Theophylline | negative |
| *Clitopilus* | Fungi | Ethyl glucuronide | positive |
| *Nigrospora* (LC, LDA score > 2) | Fungi | L-2-Hydroxyglutaric acid | negative |
| *Nigrospora* (LC, LDA score > 2) | Fungi | 2-Amino-4-methoxyphenol | positive |
| *Nigrospora* (LC, LDA score > 2) | Fungi | D-Maltose | negative |
| *Nigrospora* (LC, LDA score > 2) | Fungi | Itaconic acid | negative |
| *Nigrospora* (LC, LDA score > 2) | Fungi | Pterostilbene | negative |
| *Nigrospora* (LC, LDA score > 2) | Fungi | Phenylbiguanide | negative |
| *Nigrospora* (LC, LDA score > 2) | Fungi | Theophylline | negative |
| *Cutaneotrichosporon* (LC, LDA score > 3) | Fungi | L-prolyl-L-proline | negative |
| *Saccharomyces* (LC, LDA score > 4) | Fungi | 5,6-Dihydroyangonin | positive |
| *Alternaria* (C, LDA score > 3) | Fungi | (R)-3-Hydroxybutyric acid | negative |
| *Alternaria* (C, LDA score > 3) | Fungi | Pterostilbene | negative |
| *Coprinopsis* (C, LDA score > 2) | Fungi | Glycyrrhizin | negative |
| *Monascus*(LC, LDA score > 2) | Fungi | N-Acetyl-L-leucine | negative |
